# Aging of mesenchymal stem cell: machinery, markers, and strategies of fighting

**DOI:** 10.1186/s11658-022-00366-0

**Published:** 2022-08-19

**Authors:** Mahmoud Al-Azab, Mohammed Safi, Elina Idiiatullina, Fadhl Al-Shaebi, Mohamed Y. Zaky

**Affiliations:** 1 Department of Immunology, Guangzhou Institute of Pediatrics, Guangzhou Women and Children’s Medical Center, Guangzhou Medical University, Guangdong Provincial Clinical Research Center for Child Health, Guangzhou, 510623 China; 2 Respiratory Diseases, Shandong Second Provincial General Hospital, Shandong University, Jinan, China; 3 Department of Therapy and Nursing, Bashkir State Medical University, 450008 Ufa, Russia; 4 Molecular Physiology Division, Zoology Department, Faculty of Science, Beni-Suef University, Beni Suef, Egypt

**Keywords:** Aging, Differentiation, Mesenchymal stem cell, Cellular senescence, Senescence markers; Anti-cellular senescence

## Abstract

Human mesenchymal stem cells (MSCs) are primary multipotent cells capable of differentiating into osteocytes, chondrocytes, and adipocytes when stimulated under appropriate conditions. The role of MSCs in tissue homeostasis, aging-related diseases, and cellular therapy is clinically suggested. As aging is a universal problem that has large socioeconomic effects, an improved understanding of the concepts of aging can direct public policies that reduce its adverse impacts on the healthcare system and humanity. Several studies of aging have been carried out over several years to understand the phenomenon and different factors affecting human aging. A reduced ability of adult stem cell populations to reproduce and regenerate is one of the main  contributors to the human aging process. In this context, MSCs senescence is a major challenge in front of cellular therapy advancement. Many factors, ranging from genetic and metabolic pathways to extrinsic factors through various cellular signaling pathways, are involved in regulating the mechanism of MSC senescence. To better understand and reverse cellular senescence, this review highlights the underlying mechanisms and signs of MSC cellular senescence, and discusses the strategies to combat aging and cellular senescence.

**Graphical Abstract:**

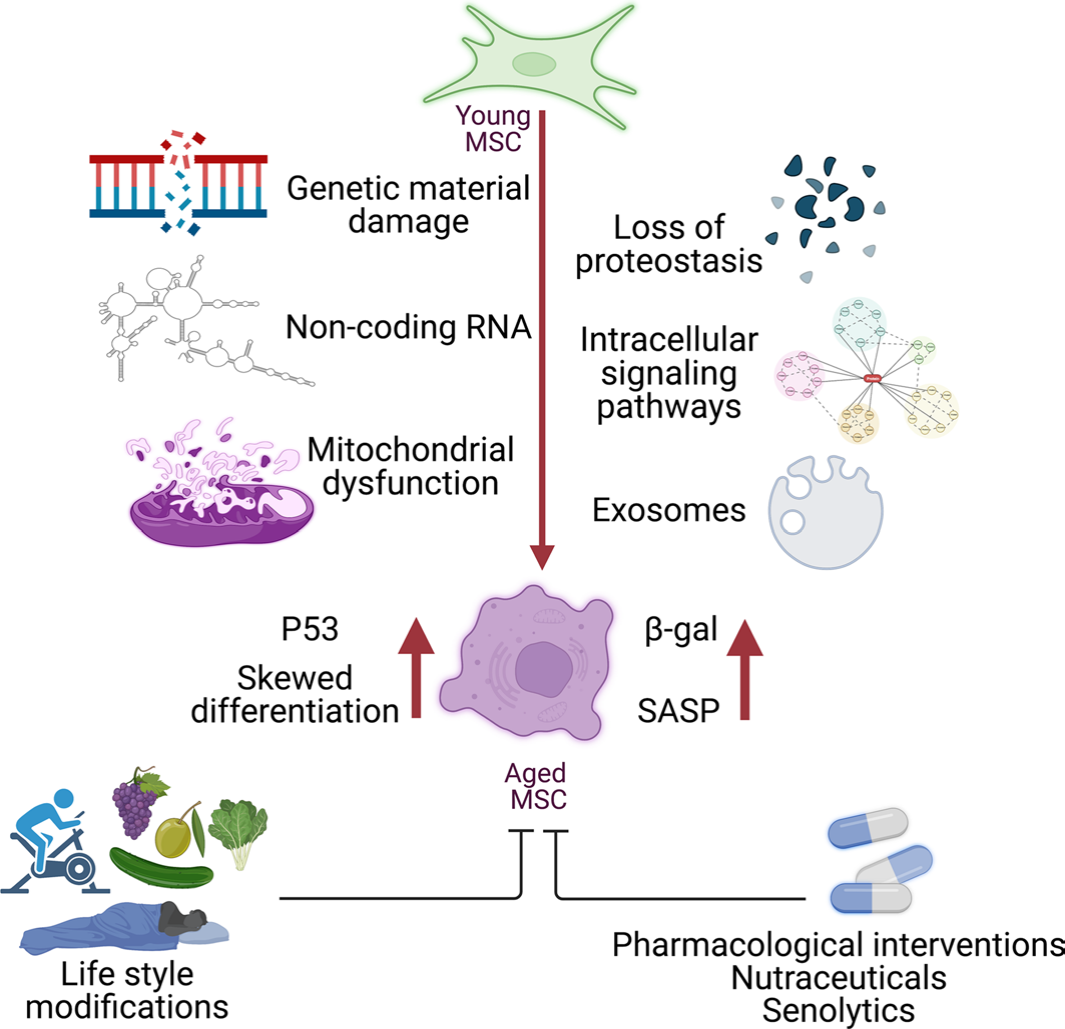

## Introduction

Mesenchymal stem cells (MSCs) are mesoderm-derived progenitor cells that have fibroblast-like morphology, adhere to a tissue culture flask, express a specific set of surface CD markers, and differentiate into osteocytes, adipocytes, and chondrocytes [1]. MSCs are cells of interest in the clinical field because of their immunomodulatory potency and capacity for tissue regeneration. Although MSCs are available from almost all adult tissues throughout the body, including adipose tissue, dental pulp, peripheral blood, and neonate-derived tissues, bone marrow remains the golden standard source for MSCs [2]. Since they were discovered in 1970 by Friedenstein, scientists characterized a variety of activities for MSCs related to their immunoregulatory power and therapeutic uses. Besides, there are numerous studies explaining various approaches to supporting MSCs’ potency in vitro and in vivo, and preventing early aging, which may interrupt their therapeutic potency. Overcoming the early aging of MSCs is becoming an issue of interest, with the aim of maintaining the optimal immunoregulatory ability of MSCs as aging can stop their vital activities. Nowadays, research in the field of cellular therapy is focused on understanding the molecular mechanisms that regulate or affect MSCs’ immunomodulatory potency, including early senescence. Therefore, understanding aging mechanisms is crucial. In addition, providing new tools that enhance MSCs’ regulation of certain biochemical mechanisms may introduce novel methods in cellular therapy. Multiple factors are involved in the aging process, including intrinsic and extrinsic factors, such as signaling pathways, cytokines, chemokines, growth factors, hormones, environmental factors, drugs, vitamins, and chemicals [3–5]. This review discusses the mechanisms and markers of MSC senescence, and how to avoid cellular senescence to enhance MSC therapeutic activity.

## Aging in general

Aging is the process of advancing toward old age, which is characterized by progressive loss of physiological functions that may lead to diseases and death. Despite early and primitive organisms, including prokaryotes, algae and protozoa, perennial plants, and some simple animals, being biologically immortal, humans, animals, and fungi undergo aging [6, 7]. Aging in humans involves an accumulation of changes over time including psychological, physical, and social changes. Aging is considered one of the greatest risk factors for most diseases, with about two-thirds of the daily death rate worldwide being due to aging-related diseases [8, 9]. Immunosenescence and inflammaging are the two main wide processes that develop with age to control the phenotypes of aging and/or aging-related diseases. They are also considered to be the underlying mechanisms that make aged people more susceptible to suffering from cancers or infections [e.g., coronavirus disease 2019 (COVID-19)]. Gut microbiota dysbiosis during aging is also involved in the process of aging through the regulation of inflammaging [10]. Although the causes of aging are not fully described, researchers claim that DNA damage, such as DNA oxidation or DNA methylation, may lead to the stoppage of the normal biological machinery [11–13].

Historically, in 1889, August Weismann was the first to theorize that aging is one part of life’s system [13]. Exploration of the relationship between caloric consumption and aging in 1934 motivated scientists to study the underlying mechanisms of aging and inflammation [14–17]. In 1952, the theory of aging by Peter Medawar was the first modern theory of aging in mammals. Medawar used the previous ideas of J.B.S. Haldane and the concepts of selection shadow. His theory was about that aging is a result of the accumulation of random mutations that occur throughout life and manifest later in life [18, 19]. In 1957, Georg C. Williams modified Medawar’s theory, stating that deaths may be caused by aging [20]. In 1977, Thomas Kirkwood proposed his aging theory, called the disposable soma theory, which is related to the limited resources consumption [21]. In 1990, after nucleic acid assays became available, scientists revealed that the aging-related genes are not random mutations, as Medawar said, but instead real genes [22]. Skulachev proposed in 1997 that gradual aging may initiate the process of evolution through survival challenges [23]. Kriete said in 2013 that the changes that come with getting older are just a way for living systems to try to stay alive and fit, even if it means getting weaker [24]. Up to now, scientists believe that aging is a biological aspect regulated and altered by a broad variety of molecular mechanisms [25].

**Fig. 1:**
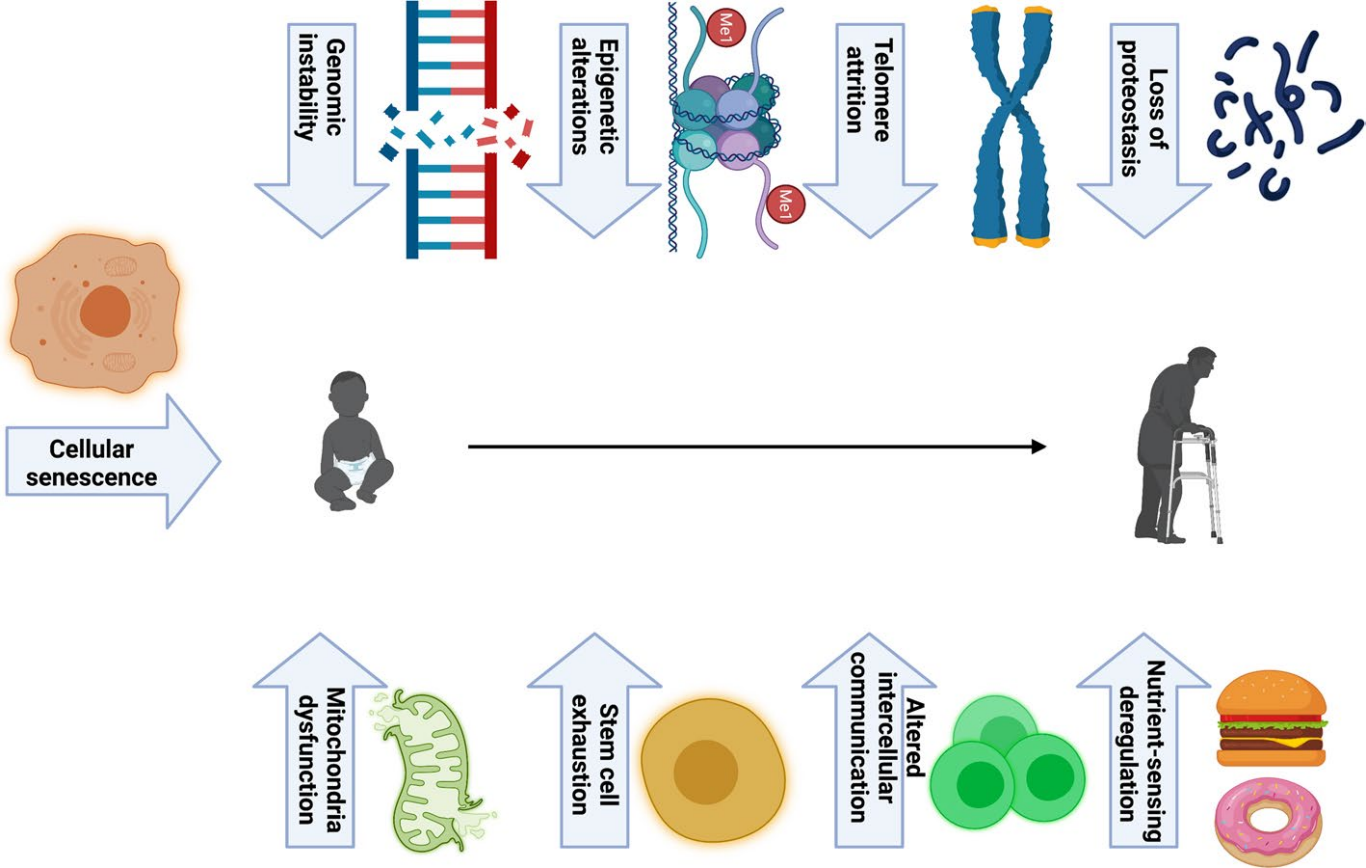
Major contributors to aging machinery. The nine hallmarks of aging: DNA damage, telomere attrition, epigenetic alteration, loss of proteostasis, mitochondrial dysfunction, cellular senescence, nutrient sensing, intracellular communication, and stem cell exhaustion [34]

There are multiple factors that contribute to the molecular basis of aging (Fig. 1). One of the most important predisposing factors of the aging process is the DNA damage caused by the accumulation of mutations, which lead to genomic instability. Reactive oxygen species (ROS), ultraviolet radiation, environmental mutagens, and chemicals are well-known agents that cause DNA damage. There is a wide range of diseases that are caused by DNA damage, including cancers, cardiovascular diseases, autoimmune diseases, and other aging-related diseases [26, 27]. Since protection of DNA integrity is the function of telomeres, their length is a factor that may regulate aging because their length decreases with age. It is reported that physical activity or exercises may support the activity of telomerase and maintain their length [28]. Not far from genetic aspects, changes in epigenetic modifications have been shown to have a contribution to stem cell aging and change their functions, especially when interacting with metabolic mechanisms. These modifications include methylation and demethylation of DNA or histone and deacetylation of histone [29]. Interestingly, the role of epigenetic alterations in aging is becoming a topic of interest owing to their reversibility, which may introduce a therapeutic method for improving life in old age and treatment of aging-related diseases, particularly cancer and cardiovascular diseases [30–33].

The processes that maintain cellular protein homeostasis or proteostasis aim to regulate protein synthesis, folding, conformation, and degradation. It is thought that these balanced mechanisms are closely linked to the aging process, especially endoplasmic stress, which disrupts proteostasis. Keeping these networks served correctly may provide promised remedies for the management of aging-related proteinopathies, such as Alzheimer’s and Parkinson’s diseases, well-known neurodegenerative disorders [35, 36]. Although nutrients are essential elements for the human body to synthesize proteins, sugars, and lipids, and get other metabolic requirements, high nutrient intake has a confirmed role in the acceleration of the aging process [14, 15]. Thus, nutrient- or energy-related signaling pathways, especially the mammalian target of rapamycin (mTOR), insulin/insulin-like growth factor 1 (IGF1), and adenosine monophosphate-activated protein kinase (AMPK) signaling systems, are among the most notable for having core roles in the regulation of aging machinery [37]. It has been reported that controlling cellular metabolism may standardize mitochondrial functions, epigenetic reactions, and energy-sensing pathways to correct the negative effects of aging [38]. On the other hand, interruptions to mitochondrial respiration and changes in intercellular communication have important contributions to the occurrence of aging. Sarcopenia, presenting as a decline in muscle mass and strength, is one of the obvious symptoms of aging. Sarcopenia is mediated by mitochondrial dysfunction that stimulates ROS generation, apoptosis, and ATP shortage leading to aging [39]. The mitochondrial theory of aging claims that mitochondrial dysfunction and oxidative stress are essential effectors in the pathogenesis of aging-related diseases, with Alzheimer’s disease as a prototype [40]. Thus, research has focused on targeting mitochondrial dysfunction and oxidative stress for the management of aging-related neuropathies [41]. Meanwhile, changes in intercellular communication including neuronal, endocrine, and neuroendocrine communication are also involved in aging. Deregulation of neurohormonal signaling leads to increased inflammation, inflammaging, and a decline in immunosurveillance, which result in life-threatening malignant and infectious diseases [25, 42]. In addition, it is known that the aging-related changes in one cell may mediate aging-related destruction in other cells [43]. More importantly, cellular senescence and exhaustion of stem cells are at the core of aging machinery. This can increase the rate of tissue aging and the decline in stem cell regenerative potential, a major characteristic of aging [25]. Thus, rejuvenation of stem cells may reverse aging-associated phenotypes [44]. In summary, the aging phenotype is a result of cellular senescence, which is due to failure in intracellular signaling homeostasis.

## Cellular senescence: MSCs as a prototype

Cellular aging is a stable cell-cycle arrest that restricts cell proliferative potential resulting from the accumulation of intercellular damage, especially oxidative stress-dependent DNA damage [45, 46]. Despite senescence being a part of the normal physiology of human cells protecting tissues from harmful malignant tumors, aging-related disease phenotypes are also thought to be merely results of cellular senescence accumulations [47, 48]. Unlike quiescent cells, which can proliferate owing to specific stimuli, senescent cells cannot reverse their proliferative activity after stimulation but remain metabolically active [49, 50]. Senescence is regulated by heterogeneous complicated pathways and predisposing factors ranging from genetic and metabolic pathways to environmental extrinsic factors. Here, we reviewed the underlying molecular mechanisms, signs of MSC senescence, and strategies of intervention as a prototype of cellular senescence.

### Mechanisms of MSC senescence

**Fig. 2:**
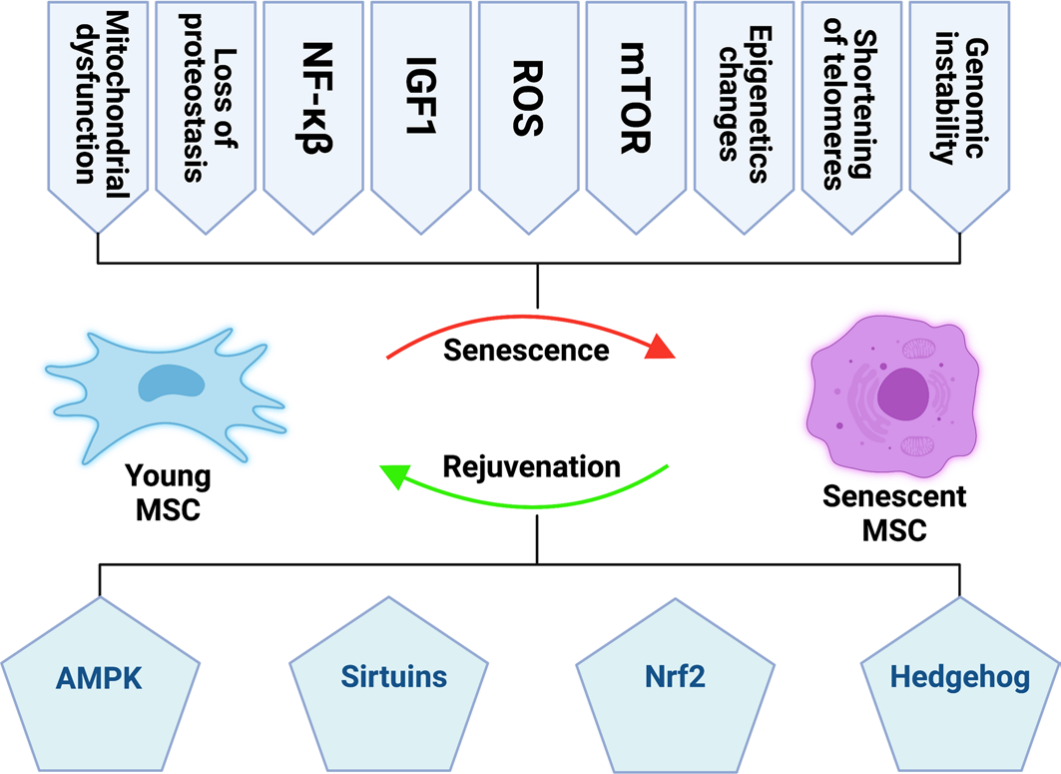
Overview of MSC senescence homeostasis. Signaling pathways, AMPK, sirtuins, Nrf2, and Hedgehog induce antisenescence effects (green), whereas signaling pathways, mTOR, ROS, IGF1, and NF-κB activate senescence (red) in MSCs. Genomic instability, telomere attrition, epigenetic alteration, mitochondrial dysfunction, and failed proteostasis induce MSC senescence

**Fig. 3:**
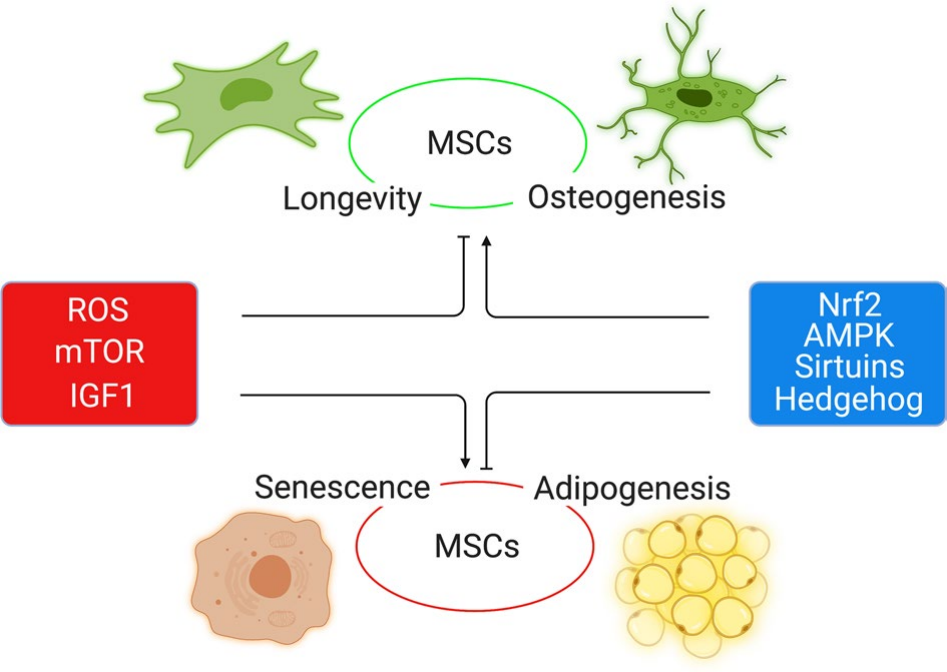
The major intracellular signaling pathways in cellular senescence and differentiation  of MSCs. AMPK, sirtuins, Nrf2, and Hedgehog work as promoters for MSC immortalization and osteogenic differentiation (green); however, ROS, mTOR, and IGF1 are considered as inducers of MSC aging and adipogenic differentiation (red). Activate (  ), inhibit (  )

Many interplaying pathways cooperate to run the aging machinery of MSCs. The major five hallmarks of MSC aging are genetic material damage, noncoding RNA and exosomes, loss of proteostasis, intracellular signaling pathways, and mitochondrial dysfunction. Herein, we tried to initiate a detailed discussion on each of them (Figs. 2, 3).

#### Genetic material damage

The first “on switch” of MSCs’ aging machinery is genomic errors. In this review, we included explanations about three processes related to genetic material damage or dysfunction, genomic instability, shortening of telomeres, and epigenetic alterations.

##### Genomic instability

Senescent MSCs are characterized by loss of their DNA repair ability and antioxidant capacity, thereby being more susceptible to tumorigenesis and DNA damage [51, 52]. There is evidence to suggest that MSCs from the late passage of human bone-marrow-derived MSCs (BMSCs) are more senescent and presented altered immunophenotype and morphology  [53]. On the other hand, oxidative stress, a higher rate of oxygen consumption, and genomic instability were all linked to MSC senescence. Thus, assessment of MSCs for percentage of aneuploidy cells before using them in clinical applications is recommended to decide how to combat MSC senescence [54, 55]. Also, in vitro propagation of MSCs attenuates their capacity for cartilage regeneration ability and presents chromosomal morphological changes associated with potential anomalous karyotypes, which accelerate premature senescence [56]. Meanwhile, in vitro expansion of Wharton’s jelly MSCs led to chromosomal changes, which may affect their clinical usage. Thus, quality-control measures should be applied before transplantation [57]. On the other hand, adipose-tissue-derived MSCs expressed low percentages of aneuploidy cells in early passages 0–4, whereas prolonged culture expansion for 5–16 passages was characterized by significantly high aneuploidy percentages without malignant transformations [58]. In addition, human embryonic stem cells maintained unstable multiple chromosomal alterations in differentiated MSCs, which enter replicative senescence after long-term culture passage [59]. Moreover, long-term in vitro expansion of MSCs showed an accumulation of γH2AX foci, a well-known marker of genomic instability whose numbers were increased in late passages with a strong increase at 16–18 passages. As a result, selecting the appropriate passage is a critical procedure before transplanting allogeneic MSCs into recipient patients, since in vitro propagations can cause MSCs to acquire genetic changes that can lead to malignant transformation [60]. Conversely, human adipose-tissue-derived mesenchymal stromal cells from the infrapatellar fat pad of patients with osteoarthritis showed genomic stability even after long-term in vitro passages. These genomic assessment assay findings revealed no telomere attrition, telomerase activity, or microsatellite instability associated with sustained expression of incompatibility repair genes [61]. In accordance with these findings, Scheer and his coworkers found that human umbilical cord matrix stem cells  expansion in vitro does not cause any genetic changes including karyotyping, telomerase mechanisms, and cell-cycle-regulating genes, nor was tumorigenesis detected after injection in immunocompromised mice [62]. These findings introduce evidence regarding the safety of the therapeutic use of MSCs. In other words, although genomic instability consequence MSC aging, it is still unclear whether in vitro expansion is a cause.

##### Shortening of telomeres

Telomeres are repeated nucleotide sequences placed at the ends of each chromosome that prevent their destruction and adhesion with neighboring chromosomes. Owing to their anatomical location, they are more susceptible to deterioration caused by DNA damage accumulation through age. Accordingly, polymerases of DNA undergoing replication are characterized by a decreased ability to synthesize a complete end of linear DNA [63, 64]. Hayflick limit, the famous indicator used for detecting the maximum limit of cell culture passages in vitro, is characterized by telomere exhaustion that leads to restricted proliferative capacity [65, 66]. Telomere attrition regulates MSC senescence through activation of downstream signaling of oncogene suppressor protein p53 and attenuation of metabolic activity of mitochondria through peroxisome proliferator-activated receptor gamma (PPARγ) co-activator 1α/β (PGC-1α/β) [67]. Even though the MSCs at the Hayflick limit are suspected to get telomere attrition, pluripotent stem cells do not experience deterioration in telomeres [68]. Sublethal prolonged doses of hydrogen peroxide induced senescence of MSC and associated with telomeres attrition after 4 weeks [69]. In addition, progeroid syndromes, which are still a subject of debate regarding their relation to accelerated aging, also experience telomere attrition [70]. On the basis of this background, researchers have attempted to identify drugs that may be able to maintain the length of telomeres in MSCs but, unfortunately, thus far have had no success. Four drugs were studied: navitoclax, danazol, quercetin, and nicotinamide riboside [71]. Despite Werner syndrome-derived lineage-specific stem cells being characterized by premature senescence, after reprogramming, scientists successfully protected them from aging through the elongation of telomeres [72]. Also, vitamin C was able to reverse a variety of senescence features, including telomere attrition [73]. Meanwhile, estradiol 2 (E2) reduces MSC and chondrocyte senescence in premenopausal women through a telomere length-dependent manner [74]. Taken together, whereas telomerase stimulation can decelerate aging in experimental animals [25, 75], telomere attrition can occur physiologically, but pathologically, these attritions may accelerate aging in mammals and MSCs.

##### Epigenetic changes

Epigenetic changes are genetic modifications that do not involve changes in DNA nucleotide sequences. The breakdown in the homeostasis of epigenetic modifications is critically suggested in MSC senescence. In this context, MSCs derived from normal and fetus-affected pregnancy amniotic fluid showed alteration in repressive markers of histone, *EZH2*, SUZ12, and BMI, and chromatin modifiers DNMT1 and HDAC1 [76]. In addition, DNA hyper-hydroxymethylation associated with 5mC loss in late age may lead to epigenetic alterations in MSCs affecting DNA methylation over a lifetime. Thus, the age of the bone marrow donor should be considered for appropriate and safe transfusion procedures [77]. Meanwhile, epigenetic assessment by pyrosequencing for BMSCs derived from patients with myelodysplastic syndrome or myeloid leukemia, and healthy controls, revealed that MSCs from patients had hypomethylation compared with those from healthy controls [78]. Also, placental MSCs revealed a tendency to deposit methylation modification after starting in vitro expansion. Thus, it is necessary to study epigenetic alterations prior to clinical usage [79]. Franzen and his group discovered that the DNA methylation changes that are associated with MSC senescence are not synchronously co-regulated, but they occur in a highly reproducible way. Seemingly, they may be stochastically produced by some epigenetic changes [80]. Thus, the epigenetic profiling of MSCs prior to therapeutic use remains a critical issue [81]. Conversely, flow-up of epigenetics within MSCs from amniotic fluid (AF-MSCs), amnion membrane (AM-MSCs), endometrium (EM-MSCs), and Wharton’s jelly MSCs showed that AF-MSCs, AM-MSCs, and EM-MSCs had constant expression pattern of H19, while variable expression of H19 was observed in WJ-MSCs [82]. This suggests that amniotic-fluid-derived MSCs  could be a favorable type of MSC for cellular therapy owing to their relative epigenetics stability. On the other hand, epigenetic silencing of HDAC9c may associated  by induced expression of EZH2, promoted osteogenesis at the cost of adipogenesis, and the involvement of the PPARγ pathway [83]. This provides a promised therapeutic target that may improve the treatment of patients with osteoporosis. More importantly, the paracrine activity of MSCs' senescence-associated secretory phenotype (SASP) could be linked to epigenetic changes that support the senescence status. For example, monocyte chemoattractant protein 1 (MCP1), a predominant chemokine secreted in SASP of MSCs, was reported to be regulated epigenetically  by BMI1 then through its cognate receptor, chemokine (C–C motif) receptor 2 (CCR2), to induce senescence by stimulating oxidative stress that then activates the p53/p21 pathway through the p38–MAPK signaling system [84]. In conclusion, maintaining the steady epigenetic state of MSCs is a crucial step in preventing their senescence.

#### Noncoding RNA and exosomes

As nontranslated RNA is very important in the regulation of multiple mechanisms in cellular machinery related to genomic stability, it can also contribute to the modulation of MSC aging. Recently, there have been many reports explaining the importance of noncoding RNA in the aging and differentiation of MSCs [85, 86]. For example, miRNA-155-5p is elevated in human serum and MSCs of aged donors but not in young donors. Noncoding RNA miRNA-155-5p induces MSC senescence through mitochondrial dysfunction in an AMPK-dependent way. Inhibition of miRNA-155-5p compromised cardiac impairment in an aged mouse model, indicating a new target to rejuvenate MSCs [87]. In addition, microRNAs are differentially expressed by MSCs and regulated by MSCs’ SASP. Indeed, they can interact with aging-related pathways, making them an interesting therapeutic target in aging-related diseases and MSC senescence [88]. More importantly, noncoding RNA as part of MSCs’ SASP can be secreted in exosomes to induce cellular senescence in young cells. It is believed that noncoding RNA may have a role in linking cellular senescence with aging-related diseases [89–91].

Use of MSC exosome therapy in regenerative medicine for aging-related diseases is being developed at the level of preclinical research and sometimes at the clinical level [92]. Thus, it is critical to discuss the relationship between the aging machinery of MSCs and their exosomes to allow translation of this research to the field of clinical medicine. It is also important to note that noncoding RNA and MSC aging is usually discussed in association with exosomes owing to noncoding RNA being one of the important ingredients of exosome vesicles. Exosomes play a dual critical role in aging and cellular senescence in both directions, inducing aging in case of SASP or having an anti-aging effect if secreted by young and healthy MSCs [93–95]. Exosomes are able to regulate aging of MSCs because they contain a variety of immunomodulatory mediators, including noncoding RNA. Briefly, noncoding RNA is at the core of MSCs’ aging modulation, either intracellularly or by paracrine exosome secretions in the neighboring niche.

#### Loss of proteostasis

Proteostasis is a collection of processes that regulate the correct synthesis, folding, trafficking, aggregation, disaggregation, and configuration of protein. Protein synthesis is a vital system that is an essential mechanism for all viable organisms as well as longevity in many organisms [96, 97]. It has been reported that interruption of physiological protein homeostasis contributes to the pathogenesis of a variety of diseases, such as diseases caused by loss of protein function, e.g., cystic fibrosis, and diseases of protein toxic functions, e.g., Alzheimer’s, Parkinson’s, and Huntington’s diseases. Proteostasis is disturbed by genetically misfolded proteins, and/or environmental stress that leads to an imbalance in folding capacity, degradation, and aggregation. Therefore, molecular interventions to support the process of proteostasis by therapeutic chaperones or enhancing proteostasis efficiency by some regulators may be introduced in innovative ways in the treatment of aging-related diseases and other proteinopathies [98, 99]. For example, FOXO, which has a role in cellular transcription through its target 4E-BP, contributes to the removal of accumulated damaged proteins that interrupt proteostasis, thereby increasing lifespan and improving aging symptoms [100]. In addition, activation of the transcription factor HSF-1, which has well-known regulatory activity on heat-shock responses, may have a role in immortalization in eukaryotes. This activation regulated by insulin/IGF-1-like signaling (IIS) through DDL1 and DDL2 regulators [101]. Moreover, Gehrig and his team declared that stimulation of *heat shock protein 72 (Hsp72)* by pharmacological stimulator BGP-15 improved the symptoms of Duchenne muscular dystrophy in two mouse models and enhanced longevity. BGP-15 regulated the removal of intracellular calcium by relieving the stressed sarcoplasmic/endoplasmic reticulum calcium-Ca(2+)-ATPase (SERCA) through activation of *Hsp72*, which stimulated the correct function of *SERCA* to decrease muscle destruction [102]. In MSCs, the proteasome is an important candidate that preserves proteostasis, and its dysfunction leads to undesired biological changes including MSC senescence. Although the role of the proteasome in MSC aging and potency still not fully explained, Kapetanou and his co-workers discovered that the proteasome is closely related to MSCs’ immunomodulatory function and aging. They found that proteasome activity is decreased in aged human MSCs derived from Wharton’s jelly and adipose tissues. The expression of proteasome β-2/5 promoter region may increase after being connected by oct4, which may provide a novel procedure to enhance MSCs’ stemness and lifespan [103]. It is reported that proteostasis of transcription factor 7-like 2 (TCF7L2) may have an important role in MSCs’ stemness regulation [104]. An excellent discussion of proteostasis and its effects on MSCs’ stemness was reviewed in this publication [105]. In summary, stable proteostasis in MSCs may keep the cells away from cellular senescence.

#### Intracellular signaling pathways

Signal transduction is the process by which cellular functions are performed through the transmission of signals by a series of molecular reactions. The components of this process are a variety of molecules and receptors that orchestrate all biological cellular cascades as well as the three events of the central dogma: DNA replication, mRNA transcription, and protein translation. Among these is cellular senescence, which is also regulated by intracellular signaling pathways [37]. Here, we discussed the roles of mTOR, ROS, IGF1, AMPK, sirtuins, Hedgehog, and other signaling pathways related to the aging of MSCs (Figs. 2, 3), as they all have interaction with the p53 pathway, a major player in cellular senescence.

##### mTOR

mTOR is a nutrient-sensing signaling pathway that works to detect high levels of amino acids [106]. Both mTOR complexes, TORC1 and TORC2, participate in the kinase activity of mTOR. It is known that mTOR signaling is implicated directly in inducing aging in unicellular organisms up to highly differentiated organisms, and its dysregulation is involved in aging-related diseases [107, 108]. Thus, inhibition of the mTOR pathway by rapamycin induced longevity in eukaryotes [109, 110]. Additionally, the senescence of MSCs associated with severe aplastic anemia in mice is attributed to increased mTOR expression, which stimulated increased levels of senescence-associated beta-galactosidase (SA-β-gal) [111]. Also, rapamycin ameliorated signs of senescence in MSCs from patients with systemic lupus erythematosus (SLE) and enhanced the immunomodulatory potency of MSCs from MRL/lpr mice through inhibition of mTOR signaling pathways [112]. As well, inhibition of mTOR-stimulated autophagy by melatonin protected MSCs from *p*-cresol-stimulated senescence through inhibition of oxidative stress accumulation [113]. Although it is still not fully explained how glucose induces cellular senescence, increased levels of phosphorylated phosphatidylinositol 3-kinase-protein kinase B (Akt) and phosphorylated mTOR expression were observed among cells treated with high glucose. More importantly, the inhibition of Akt reversed senescence in the presence of high glucose. These findings suggest that glucose-induced senescence in MSCs is mediated by the Akt/mTOR signaling pathway [114]. d-Galactose is a sugar also reported to stimulate MSC senescence through stimulating ROS generation. Zhang and his colleagues found that giving coenzyme Q10 to MSCs that had already been treated with d-galactose may reduce the expression of AKT and mTOR and lower the number of senescent MSCs. This confirmed that coenzyme Q10 rejuvenates senescent MSCs through inhibition of the Akt/mTOR signaling pathway [115]. Thus, it appears that, as high caloric intake induces aging, high glucose uptake also induces MSC senescence. In addition, aged MSCs are characterized by attenuated autophagy, but the cause is still unclear. Interestingly, reduction of IGF1 levels by gene silencing has been observed to have antisenescence activity on MSCs of bone marrow through inducing autophagy, thereby downregulating the Akt/mTOR axis [116]. Meanwhile, a novel tryptophan metabolite, 5-methoxytryptophan (5MTP), was reported to have potential effects in reversing high glucose, and a sublethal dose H_2_O_2_-induced senescence of BMSCs. It is believed that 5MTP exerts its antisenescence effects through enhancement of FoxO3a expression, and elevation of superoxide dismutase, but conversly induced *mTOR* [117]. Also, inhibition of microRNA-188, an aging accelerator factor, contributed to improvement of the biased differentiation of MSCs and introduced a promising strategy to avoid age-related bone loss. MicroRNA-188 stimulated accumulation of fat in the bone marrow and decreased numbers of osteoblasts through targeting RPTOR-independent companion of mTOR complex 2 and histone deacetylase 9. Stimulation of MSC adipogenic differentiation came at the cost of osteogenic differentiation, which led to bone loss diseases such as osteoporosis [118]. Moreover, mTOR/Pl3K axis interruption has been reported as a successful procedure to enhance osteogenesis in diseases with defective bone synthesis. For example, inhibition of the mTOR/Pl3K pathway was associated with reduced mitochondrial dysfunction. The authors believe that this anti-aging phenotype was regulated by increasing mitophagy via knockdown of leucine-rich repeat containing 17 [119]. Furthermore, use of antiresorptive nitrogen-containing bisphosphonate, zoledronate, in patients with osteoporosis yielded amazing results by extending the patients’ survival [120]. This effect was also observed in MSCs, where zoledronate preserved their proliferation and differentiation capacity through decreasing accumulation of DNA damage, the underlying mechanism of MSC aging. These effects were mediated by inhibition of the mTOR signaling pathway [121]. Meanwhile, the reduction of estrogen-related receptor α (ERR α) and mitochondrial glutaminase associated with aged MSCs induced impairment of osteogenesis differentiation. However, mTOR may regulate mitochondrial glutaminase activity and ERR-α antagonist, leading to relief of the osteogenesis differentiation impairment of human MSCs [122]. Interaction of Indian Hedgehog signaling with mTOR has been shown to potentially regulate senescence of BMSCs through modulation of downstream substrates of TORC1/2 complexes 4EBP1 and p70S6K12 [123]. In conclusion, the mTOR signaling pathway is a key regulator in MSC senescence owing to its interaction with the autophagy system and ROS signaling.

##### ROS

ROS are chemicals containing oxygen, such as peroxide, superoxide, and hydroxyl groups, accumulated in cells owing to the normal metabolism of oxygen. Although ROS are involved in cellular signaling to perform physiological functions, they are also involved in different pathological mechanisms. Oxidative stress is caused due to increased ROS generation and decreased antioxidant molecules in animals and plants. ROS production is known to be one of the major contributors to the regulation of aging and predisposition of aging-related diseases [124–126]. Also, increased oxidative stress after in vitro expansion of MSCs is associated with decreased immunomodulatory function. This phenotype is characterized by suppressed proliferation and decreased expression of some surface antigens, including CD13, CD29, and CD44. In addition, ROS decreased MSCs’ ability to suppress immune cells such as T cells. In other words, oxidative stress is one of the major events associated with replicative senescence of MSCs, which restricts the number of passages and cell potency [127]. Indeed, MSCs derived from adipose tissue of elderly people are characterized by increased oxidative stress of mitochondrial origin compared with MSCs of younger people. MSCs from elderly people also have a decreased ability to form colonies, contain elevated percentages of SA-β-gal-positive cells, and exhibit upregulated P21. Moreover, MSCs from aged persons experience reduced potential in adipogenic and osteogenic differentiation and restricted migration capacity associated with decreased expression of chemokine receptors CXCR4 and CXCR7 [128]. ROS are also linked to Rho family GTPase Cdc42, which plays a role in stimulating MSC senescence. For example, MSCs from rat adipose tissue of advanced age show senescence signs including oxidative stress as well as decreased growth ability and differentiation capacity into osteogenic, adipogenic, and chondrogenic cells. However, inhibition of Cdc42 by CASIN decreased the generation of ROS, level of  p16^Ink4a^, and activity of ERK1/2 and JNK signaling pathways, and increased the differentiation potential toward adipogenic and osteogenic pathways [129]. As MSC aging limits their therapeutic use, scientists believe that understanding ROS-related signaling pathway mechanisms that support replicative senescence of MSCs may contribute to the improvement of cellular therapy procedures. Nowadays, MSC research is focusing on how to delay or reverse MSCs’ replicative senescence, and oxidative stress is one of the major related issues. Therefore, extensive efforts are being made to interrupt the senescence activity of ROS on MSCs. The hormone of the pineal gland, melatonin, is reported to regulate ROS generation, thereby promoting the general physiological function and immunomodulatory potency of MSCs [130]. Meanwhile, a milk iron-binding glycoprotein, lactoferrin, was noted to rejuvenate human MSCs through its antioxidant activity. Treatment of human MSCs by lactoferrin after being senescent owing to exposure to hydrogen peroxide revealed that lactoferrin suppressed hydrogen-peroxide-induced intercellular ROS and apoptosis. This indicates a promising role for lactoferrin as an antioxidant and enhancer for immunomodulatory potency of MSCs to prevent the senescence effect caused by ROS [131]. Treatment of MSCs with nicotinamide enhances their proliferative and multilineage differentiation potential. It is thought that nicotinamide’s mode of action is related to its ability to decrease ROS [132]. Also, inhibition of NADPH oxidasNox2, a well-known source of intracellular ROS, by acetovanillone or Nox2 silencing contributed efficiently to enhancing the antisenescence and anti-apoptotic activity in BMSCs, thereby promoting their therapeutic power in the treatment of myocardial infarction. In the same context, blocking of Nox2 increased cell viability, improved senescence markers induced by H_2_O_2_, and decreased apoptotic cells as well as restricting ROS accumulation and expression of p-p53, p21, p-FoxO1, and Bax proteins. Interestingly, Nox2 overexpression amplified senescence, decreasing viability and apoptosis of MSCs [133]. Additionally, ginsenoside Rg1 increased lifespan, proliferation, and colony formation of bone-marrow stromal cells. Incubation of bone-marrow mononuclear cells with ginsenoside Rg1 caused a decrease in SA-β-gal positive cells and apoptotic cells associated with suppressed ROS generation and enhanced colony-forming ability [134]. Moreover, hydrogen has an anti-aging effect through decreasing oxidative stress in rat MSCs after administration of hydrogen-rich saline. This antioxidant activity is associated with more efficient trilineage differentiation power and a decrease in the accumulation of cells at G1 phase in the cell cycle. More importantly, hydrogen-rich saline reduced the expression of aging-associated proteins p53 and p21 [135]. Furthermore, amelioration of ROS-mediated oxidative stress in MSCs is now possible by a variety of procedures, including exposure to basic fibroblast growth factor (bFG*F*), Ex-4 preconditioning, and pigment epithelium-derived factor (PEDF). In addition, extracellular matrix components play a role in promoting MSCs’ self-repair and correct differentiation via increasing expression of enzymes with antioxidant activities that neutralize the elevated levels of ROS. Pharmacologically, N-acetyl cysteine (NAC), NAC and l-ascorbic acid 2-phosphate, and preconditioning with vitamin E, metformin, fullerol, fucoidan, carvedilol, nicorandil, and 5-azacytidine are all effective antioxidant candidates that reduce ROS-induced senescence of MSCs [136]. Inhibition of ROS signaling by diphenyleneiodonium chloride (DPI) and NAC has been demonstrated to decrease Indian hedgehog (IHH) depletion-induced senescence of BMSCs. Indeed, blocking of ROS contributed to correcting biased differentiation and restricting aging-related genes and signaling pathways [123]. In other words, oxidative stress can induce MSC senescence; however, there are a variety of antioxidants that can prevent oxidative stress-induced senescence.

##### IIS

IIS is one of the most important signaling pathway among the nutrient-sensing pathways that are downregulated by nutrient restriction and contribute to the regulation of aging. Literature evidence indicates the important effect of IIS reprogramming on the regulation of pathways closely related to aging and longevity, such as Akt, FOXO, mTOR, and AMPK [137, 138]. For example, IGF1-binding protein 4 (IGFBP-4) stimulates senescence of MSCs obtained from rat bone marrow, which is characterized by proliferation depletion. Proliferation suppression mediated by IGFBP-4 is restored after adding IGF1 receptor antagonist [139]. Meanwhile, Wu and his team found that IGFBP-4 expression increased with age in rat BMSCs and contributed to osteogenesis impairment. They reported that alkaline phosphatase activity, osteoblast marker genes, and calcium deposition were also restricted in parallel with IGFBP-4 overexpression [140]. Also, IGF1-binding protein 5 (IGFBP-5) was observed as a trigger for cellular senescence through stimulation of cell-cycle accumulation at G0/G1 phase and upregulation of tumor suppressor p53 expression [141]. Additionally, IGF1-binding protein 4/7 (IGFBP-4/7) in the conditioned medium of senescent MSCs induced the senescence of young MSCs and increased the percentage of apoptotic cells. Further exploration showed that inhibition of IGFBP-4/7 reversed senescence and apoptosis of MSCs [142]. On the other hand, MSCs of human umbilical cord Wharton’s jelly from mothers with gestational diabetes mellitus revealed impaired proliferation, differentiation, stemness, mitochondrial function, and upregulation of cell-cycle inhibitors, p16^Ink4a^, p21, and p27. Such findings indicate that hyperglycemia can stimulate the aging of MSCs and attenuate their stemness through the interruption of insulin and its downstream pathways [143]. Indeed, insulin-resistance-induced senescence of MSCs from adipose tissue of horses with equine metabolic syndrome was suppressed after exposure to *Spirulina platensis* in vitro. In vivo, feeding a horse with supplementary *Spirulina platensis* caused weight loss and ameliorated signs of senescence. These results suggest the promising therapeutic potentials of *Spirulina platensis* in increasing MSCs’ stemness and in use as a treatment of aging-related diseases through an insulin-dependent manner [144]. Conversely, it has been reported that IGF1 and IGFBP3 play a role in osteoblastic differentiation of human BMSCs in vitro and in vivo. Scientists believe that telomerase activity contributes to the upregulation of IGF1 signaling proteins, which in turn stimulates Akt phosphorylation and amplifies the activity of alkaline phosphatase. These findings support the theory of positive action of IGF1 in osteogenesis and longevity [145]. Taken together, it is very clear that high caloric intake induces MSC senescence through upregulation of IIS.

##### AMPK

AMPK is one of the major players in energy homeostasis of the cell, which can be stimulated in a state of low energy through the identification of elevated AMP. It plays an important role in helping the cell to use and oxidize sugar and lipids, thereby protecting the cell from the adverse activities of increased glucose and fat. AMPK is considered as a part of catabolic nutrient-sensing pathways that may have a positive effect on longevity through downregulation of mTOR signaling [146–148]. In patients with neurodegenerative diseases, such as amyotrophic lateral sclerosis, MSCs revealed signs of senescence compared with normal control of MSCs from bone marrow. Further assays on such cases revealed that the AMPK pathway was downregulated but upregulated after treatment by resveratrol [149]. Meanwhile, the AMPK pathway was reported as an effector in suppressing oxidative stress to support osteogenic differentiation of MSCs in a melatonin-dependent manner. In other word, melatonin is promised to enhances osteogenesis in patients with osteoporosis through activation of the AMPK pathway, FOXO3a, and RUNX2, which are inhibited by ROS generation [150]. Also, it is reported that C1q/tumor necrosis factor-related protein 9 (CTRP9) has an anti-aging effect in MSCs through activation of the AMPK pathway. Additionally, knockdown of AMPK inhibited the rejuvenation activities of CTRP9 on MSCs from aged mice by increasing oxidative response [151]. Interestingly, an AMPK activator, AICAR, is already proven to have an obvious effect in the treatment of mouse interstitial fibrosis of the aged heart. This therapeutic activity is attributed to the activation of the AMPK pathway in mesenchymal fibroblasts, a progeny of MSCs, which decrease the activity of the Erk pathway, decreases MCP1 production, and discourages infiltration of leukocytes, thereby interrupting fibroblast formation [152]. This may introduce a promised therapeutic strategy for interstitial fibrosis which is a component of heart failure among elderly people. In addition, macrophage migration inhibitory factor (MIF) is reported as an anti-aging factor in MSCs from mice through increased phosphorylation of AMPK by CD74. Additional assays revealed that silencing of the AMPK pathway interrupts the antisenescence activities of MIF on MSCs [153]. Furthermore, Chen et al. reviewed the contributions of AMPK in osteogenic and adipogenic differentiation of MSCs, a powerful sign of MSC senescence. They reported that AMPK promotes osteogenesis at the cost of adipogenesis in order to suppress the replicative senescence of MSCs. Although it is not clear how AMPK corrects the biased differentiation of MSCs, it is reported that AMPK may exert its anti-aging activity through Erk activation [154], mTOR inhibition, and activation of Wnt/beta-catenin pathways [155]. Collectively, AMPK is a major pathway that has antisenescence effects in MSCs.

##### Sirtuins

Sirtuins are also referred to as “silent mating type information regulation 2 homolog 1” (SIRT1). SIRT1 is one of the nutrient-sensing pathways upregulated in association with caloric restriction and works in an enzymatic manner to deacetylate cellular proteins in order to support cellular nutrient consumption. It is one of the well-known genes that contributes to longevity along with the AMPK pathway through increasing insulin sensitivity and inhibiting pathways that enhance senescence, such as p53 [156]. It is known that sirtuin homologs SIRT3, and SIRT4 may promote longevity of *Saccharomyces cerevisiae* through inhibiting simultaneous  coexpression of a and α mating-type genes [157]. Also, MSCs from aged rat bone marrow revealed decreased expression of nicotinamide phosphoribosyl transferase (Nampt)  and SIRT1 gene [158]. Another study showed that SIRT3 expression is attenuated with in vitro expansion-induced replicative senescence in MSCs. Further exploration showed that deletion of SIRT3 led to the inhibition of differentiation capacity of MSCs into osteocytes and adipocytes. In addition, overexpression of SIRT3 reduced ROS generation and restored the differentiation ability of senescent MSCs [159]. Indeed, overexpression of SIRT1 in MSCs of mice prevented bone loss, which was associated with increased bone formation, osteoblast count, alkaline phosphatase activity, and osteogenesis-related genes. Moreover, increased expression of SIRT1 in MSCs of an osteoporosis mouse model showed increased longevity, bone growth, and decreased osteoclastogenesis. The osteogenic promotion by SIRT1 is attributed to a reduction of forkhead box O3a (FOXO3a) acetylation and increased expression levels of *superoxide dismutase 2 (SOD2)* and *FOXO3a* [160]. Moreover, MSCs reversed the DOXO-induced senescence of cardiac cells through SIRT1, which inhibits microRNA-34a. The study showed that SIRT1 limited the expression of p16^Ink4a^ and p53 and promoted telomerase activity and telomere length [161]. Furthermore, decreased expression of SIRT1 and SIRT3 has been proven to be responsible for the senescence of elderly adipose tissue MSCs. Meanwhile, SIRT1 overexpression reduced the activity of the p53/p21 pathway in order to regain the MSCs’ normal differentiation ability and decrease aging [162]. Collectively, almost all mammalian sirtuins (SIRT1-7), but especially SIRT1-3 and SIRT6, play core roles in maintaining and modifying MSCs’ cellular protein after transcription, particularly deacetylation. These sirtuin activities regulate mitochondrial machinery, including respiration and protection from oxidative stress, as well as MSC differentiation and paracrine secretions [163]. Despite the variety of reports revealing the direct role of sirtuins in MSC differentiation, Zainabadi showed that SIRT1 may promote MSC differentiation through specific lineages but suppress other lineages [164]. In conclusion, sirtuins play a pivotal role in cellular homeostasis to prevent MSC senescence.

##### Sirtuins and AMPK synergism

The main effector in longevity is caloric restriction through decreasing food intake, and the major pathways mediated by the low level of energy are sirtuins and AMPK. It has been reported that stimulation of sirtuins and AMPK maintains anti-aging activity and differentiation capacity of MSCs [155]. During the last decade, more attention has been paid to caloric-restriction-dependent sirtuins and AMPK synergism and its cellular interactions that alter metabolic processes and mitochondrial ROS generation to correct the biased differentiation of MSCs [155, 165, 166]. Although the mechanisms that regulate this effect remain a controversial issue, many studies have indicated the role of PGC-1α in the mediation of sirtuins and AMPK synergistic effect after deacetylation. PGC-1α not only regulates mitochondrial respiration but also regulates ROS catabolism through stimulation enzymes and nuclear receptors that scavenge ROS [155, 167, 168]. Therefore, the discovery of stimulators that induce the sirtuins and AMPK synergism may introduce an amazing procedure in outstanding therapeutic usage of MSCs without barriers of aging and skewed differentiation. Resveratrol was reported as an activator of AMPK and cellular respiration through SIRT1 [169]. Metformin is also considered a player in cellular metabolism in helping the cell to regulate glucose synthesis from noncarbohydrate sources through induction of the synergistic effect of SIRT1 and AMPK [170]. Taking together, SIRT1 and AMPK have the opposite action of mTOR and IIS pathways in senescence and differentiation machinery.

##### Hedgehog signaling

Hedgehog is an important signaling pathway involved in tissue growth and morphogenesis at the level of embryonic development. SHH, IHH, and DHH ligands, and PTCH1/2 and Smo transmembranous receptors as well as the target gene of Hedgehog, Gli, are all members of the Hedgehog signaling pathway. Scientists have suggested a promising regenerative potential for SHH in the regeneration of cardiac tissue in animals, suggesting a role for Hedgehog signaling in improving symptoms of aging-related diseases. Consistently, SHH is also reported as an anti-aging factor in aging-related neurodegenerative diseases. In vitro and in vivo findings have revealed that SHH is involved in neurogenesis, autophagy, antioxidation, and anti-inflammation [171–173]. In addition, disrupted  Hedgehog signaling in a leptin-deficiency-dependent manner regulates liver resident pericyte senescence [174]. Also, owing to its action in the rejuvenation of tumor stem cells, targeting SHH is one of the underlying mechanisms of curcumin in targeting colorectal cancer stem cells [175]. It has been reported that SHH and IHH transfection to BMSCs in vitro induces chondrogenic differentiation and prevents aging [176]. Indeed, SHH is downregulated in aged endometrium stem cells, and exogenous SHH has shown antisenescence action through regulation of SERPINB2 [177]. On the other side, Gli-1 and incompletely characterized Hedgehog homolog DHH were reported as important factors in nerve organization [178] that may protect nerves in aging-related degenerative diseases.  Moreover, Hedgehog signaling works with the IIS pathway in the opposite action to maintain the lifespan of stem cells [179]. Even though the role of Hedgehog signaling in aging remains a topic of debate, scientists believe that activation of the Hedgehog pathway may introduce an option for the treatment of osteoporosis, a well-known aging-related disease. In line with this, there are findings explaining how oxysterols exert an antisenescence effect on pluripotent mesenchymal cells through promoting osteoblastogenesis and suppressing adipocyte formation using members of the Hedgehog pathway, such as Smo receptor and Gli gene [180, 181]. IHH may modulate MSC aging and be involved in aging-related disease, such as rheumatoid arthritis [123, 182]. In addition, we showed the mode of action of this antisenescence mechanism by which IHH regulates oxidative stress and the mTOR pathway through 4EBP1 and p70S6K1/2 [123]. Indeed, we reported  increased expression of IHH in MSCs’ that may induce the immunomodulatory power after stimulation by TL1A [183]. In summary, although the role of Hedgehog pathway in MSC senescence or longevity is still incompletely understood, it is very clear that it has a considerable contribution to the process.

##### Miscellaneous signaling

The above-mentioned pathways are not the only ones that orchestrate the senescence machinery; there are also a variety of intracellular signaling pathways that contribute directly and/or indirectly to maintaining the aging status. Among those pathways are nuclear factor kappa-light-chain-enhancer of activated B cells (NF-κB)-p65, signal transducer and activator of transcription 3 (STAT3), mitogen-activated protein kinases (MAPK) or extracellular signal-regulated kinase (ERK), AKT serine/threonine kinase 1 (AKT1), and phosphatidylinositol 3-kinase (PI3k). Herein, we briefly discuss the role of each of them in MSC senescence.

**NF-κB **NF-κB is a vital signaling pathway that is present in all nucleated cells and is involved in multiple cellular responses to stimulators, such as infectious, chemical, and/or physical stimuli. Owing to its importance in the regulation of immune mechanisms, any disturbance can affect optimal function of NF-κB, leading to different diseases, such as cancer, autoimmune disorders, inflammatory diseases, and aging-related diseases [184]. The activity of the NF-κB pathway is enhanced in aged MSCs and associated with bone loss. Also, increased NF-κB expression was observed in MSCs after being stimulated by lipopolysaccharide (LPS). It is believed that targeting NF-κB activity is a promising therapeutic procedure in the treatment of aging-related bone loss [185]. In mice, enhanced NF-κB activity stimulated SASP of mesenchymal progenitors. In addition, increased NF-κB is associated with aging markers, cell cycle arrest, DNA damage, γH2AX foci, and p53, and p21 phosphorylation. Indeed, GATA4 leads to restricted osteogenesis and bone loss due to decreased osteoblast count [186]. Moreover, senescence of MSCs in eukaryotes could be inhibited by downregulation of NF-κB activity. It is reported that melatonin contributes to rejuvenating MSCs through activating Nrf2, which may inhibits NF-κB and SASP [187]. Some variable activities for NF-κB in senescence were reported with MSCs derived from adipose tissues and umbilical cord. Studies suggested a notable interaction between NF-κB and senescence-related pathway p53 [188, 189]. Recently, Hu et al. explained how BMSC senescence can be induced through nucleosome assembly protein 1-like 2 (NAP1L2) in an NF-κB phosphorylation-dependent manner [190]. Collectively, because it is the intracellular pathway that regulates SASP in MSCs, NF-κB targeting can contribute to their rejuvenation.

**STAT3** STAT3 is a well-known transcription factor that plays an important role in cellular machinery through its involvement in the synthesis of effector proteins. Nonfunctional STAT3 may lead to serious illnesses, such as cancer, rheumatic diseases, and diseases of aging [191], but regulation of JAK2/STAT3 by the humanin pathway has anti-oxidant effects [192]. The implication of the STAT3 pathway in senescence of BMSCs from patients with SLE was confirmed. For example, STAT3 upregulation is associated with increased  SA-β-gal-positive cells, disturbed cell cycle, and morphological changes [193]. More importantly, inhibition of STAT3 may provide a way to reverse senescence and treat diseases of old age. Additionally, experiments on mice unveiled that JAK2/STAT3 axis activation by leptin in MSCs may promote bone loss and delay fracture healing [194]. In the same context, senescent BMSCs from estrogen-deficient mice experienced induced JAK2/STAT3 pathway associated with SASP [195]. Though the JAK2/STAT3 pathway upregulated adipogenesis and restricted osteogenesis in a leptin-dependent manner, STAT3 may enhance MSCs’ migration ability to increase their therapeutic efficacy [196]. It is suggested that the STAT3 pathway can be involved in inducing differentiation of BMSCs into neural cells [197] and have an antisenescence role [198]. Taken together, maintaining the correct function of STAT3 is an important issue for aging homeostasis in MSCs.

**ERK** ERK or MAPK is an intracellular signaling system involved in many cellular functions, such as mitosis, meiosis, and transcription factor activation. Many extrinsic and intrinsic stimulators could stimulate ERK, including chemokines and infectious material. Dysregulated ERK underlies aging and replicative cellular senescence. One example is loss of bone formation ability, a prominent marker of MSCs’ aging. A further study uncovered that osteogenesis was inhibited owing to upregulation of ERK, which then augments ROS accumulation and decreases MSC proliferation. Also, the study stated that melatonin can reverse MSC iron overload-induced senescence through scavenging the p53/ERK/p38 pathway, thereby protecting MSCs from oxidative stress [199, 200]. Of note, ERK is involved in aging heart interstitial fibrosis, which is produced by aged MSC-derived fibroblast. Fibrosis is maintained by fibroblast secretions, collagen type 1, MCP-1, and IL-6. The transcriptional factors for these secretory proteins were regulated by farnesyltransferase (FTase)–Ras–ERK signaling [201]. In contrast, Lee et al. showed that ERK could be involved in MSCs differentiation mediated by glucagon-like peptide-1 (GLP-1). The adipogenesis markers PPRGγ, adipocyte protein 2 (AP2), and lipoprotein lipase (LPL) were suppressed by ERK axis [202]. In sum, ERK signaling plays variable roles in managing aging and differentiation of MSCs.

**AKT** Akt proteins are involved in cellular signaling, among which Akt1–3 are involved in migration, proliferation, apoptosis, and glucose metabolism. As we mentioned above, the importance of Akt in aging machinery is due to its role in the PI3K/Akt/mTOR pathway, a core effector signaling in cell-cycle regulation. In literature, the involvement of Akt in MSCs’ senescence machinery has been reported [203] in which the accumulation of ROS may contributes to the phosphorylation of Akt on IL-8 knockdown-dependent senescence in MSCs derived from the placenta [204]. On the other hand, Akt could be used by erythropoietin to protect MSCs from hyperglycemia-induced senescence through FOXOa [205]. Also, Akt activation in vitro and in vivo was involved in MSCs’ rejuvenation activity of neuron-derived neurotropic factor (NDNF), which proved to have an anti-aging effect on aged MSCs to promote the function of the injured heart [206]. Additionally, doxorubicin (DOXO)-induced MSC senescence was reversed by MIF through activation of the PI3K/Akt pathway [207]. In brief, Akt can be used as a downstream pathway that positively or negatively affects aging of MSCs.

**PI3K** PI3K is a family of enzymes that have regulatory functions in multiple cellular mechanisms, including cell viability, migration, growth, and differentiation. PI3K is an intracellular pathway that is well known to have a role in cancer. Its role in aging has also been highlighted. For example, human telomerase reverse transcriptase (hTERT) overexpression in senescent MSCs increased telomere length and telomerase activity through stimulation of PI3K/Akt pathway activity [208]. In addition, the Wnt5a/PI3K/miR-122 pathway was implicated in the mode of action of ML141, which promoted MSC hepatic differentiation through inhibition of RhoGTPase Cdc42 [209]. Moreover, mouse models of accelerated senescence with abnormal MSC immune function revealed low PI3K activity [210]. Furthermore, phosphorylation of PI3K in MSCs has been associated with the antioxidant pathway in H_2_O_2_-induced oxidative stress through increased expression of manganese superoxide mutase (MnSOD) after treatment by lycopene [211]. Conversely, targeting PI3K killed senescent cells, indicating that activation of PI3K may induce senescence [212]. Briefly, according to these publications, the role of PI3K in MSC aging still needs more exploration.

#### Mitochondrial dysfunction

Mitochondria are the respiratory organelles of eukaryotic cells that play a role in oxidative stress (OS) and reactive oxygen species (ROS) production, as well as adenosine triphosphate (ATP) production, through the mitochondrial respiratory chain (MRC). Five enzymatic complexes (I–V) of integral membrane proteins are involved in MRC: NADH–CoQ reductase (complex I), succinate–CoQ reductase (complex II), CoQ–cytochrome c reductase (complex III), cytochrome C oxidase (complex IV), and ATP synthase (complex V) . MRC interruption can lead to mitochondrial dysfunction, which contributes to the oxidative stress in MSCs and can increase apoptosis. There is accumulating evidence explaining how mitochondrial dysfunction and mitochondrial ROS can affect the aging process in MSCs [213, 214–216]. An association between accelerated senescence of MSCs and mitochondrial dysfunction has been reported. Studies reported increased levels of mitochondrial ROS and decreased antioxidant levels in senescent MSCs. For example, accumulation of mitochondrial free radicals due to SOD2 deficiency leads to suppression of differentiation power into osteocytes or adipocytes. It is reported that the underlying mechanism in such a case is an increasing amount of alpha-ketoglutarate in SOD2-deficient MSC precursors [217]. Also, senescent BMSCs from a patient with idiopathic pulmonary fibrosis revealed significant mitochondrial dysfunction associated with DNA damage accumulation and critical defect in MSCs’ stemness. In addition, senescent MSCs with mitochondrial dysfunction have the ability to induce aging in normal fibroblasts, suggesting that idiopathic pulmonary fibrosis could be linked with aging of MSCs [218]. Indeed, DNA hypomethylation of mitochondrial origin was considered a marker for senescence in MSCs derived from the human fetal heart [219]. Also, the niche from which MSCs were derived is an important issue, with MSCs being reported as a promoter of some diseases of aging. Kornicka et al. reported that MSCs extracted from patients with metabolic syndrome and type 2 diabetes mellitus are characterized by senescence signs, particularly mitochondrial deterioration [220]. Moreover, umbilical cord MSCs from mothers with gestational diabetes mellitus also displayed early aging with mitochondrial dysfunction associated with depleted cellular function and respiration [221]. Interestingly, it is suggested that  bone of patients infected with human immunodeficiency virus (HIV) are characterized by aging phenotype. The experiments showed that HIV proteins Tat and Nef enhanced mitochondrial dysfunction and inhibited MSCs differentiation into osteoblasts, suggesting that patients with HIV are more susceptible to bone loss and osteoporosis [222]. Furthermore, MSCs in the immune thrombocytopenia niche revealed senescence signs including decreased mitochondrial membrane potential. More importantly, the researchers indicated the possibility of using platelet-derived growth factor (PDGF) to protect MSCs of patients with immune thrombocytopenia [223]. Meanwhile, treatment of mitochondrial deteriorated MSCs with 5-azacytidine (5-AZA) DNA methyltransferase inhibitor restored their therapeutic capacity, as indicated by increased proliferation rate, decreased ROS accumulation, increased SOD activity, and ameliorated apoptosis [224]. Therefore, understanding the mechanisms that cause mitochondrial dysfunction in MSCs is critical in aging-related diseases as this may enable the introduction of a novel therapeutic target and add to our comprehension of senescence mechanisms.

### Senescence markers in MSCs

**Fig. 4:**
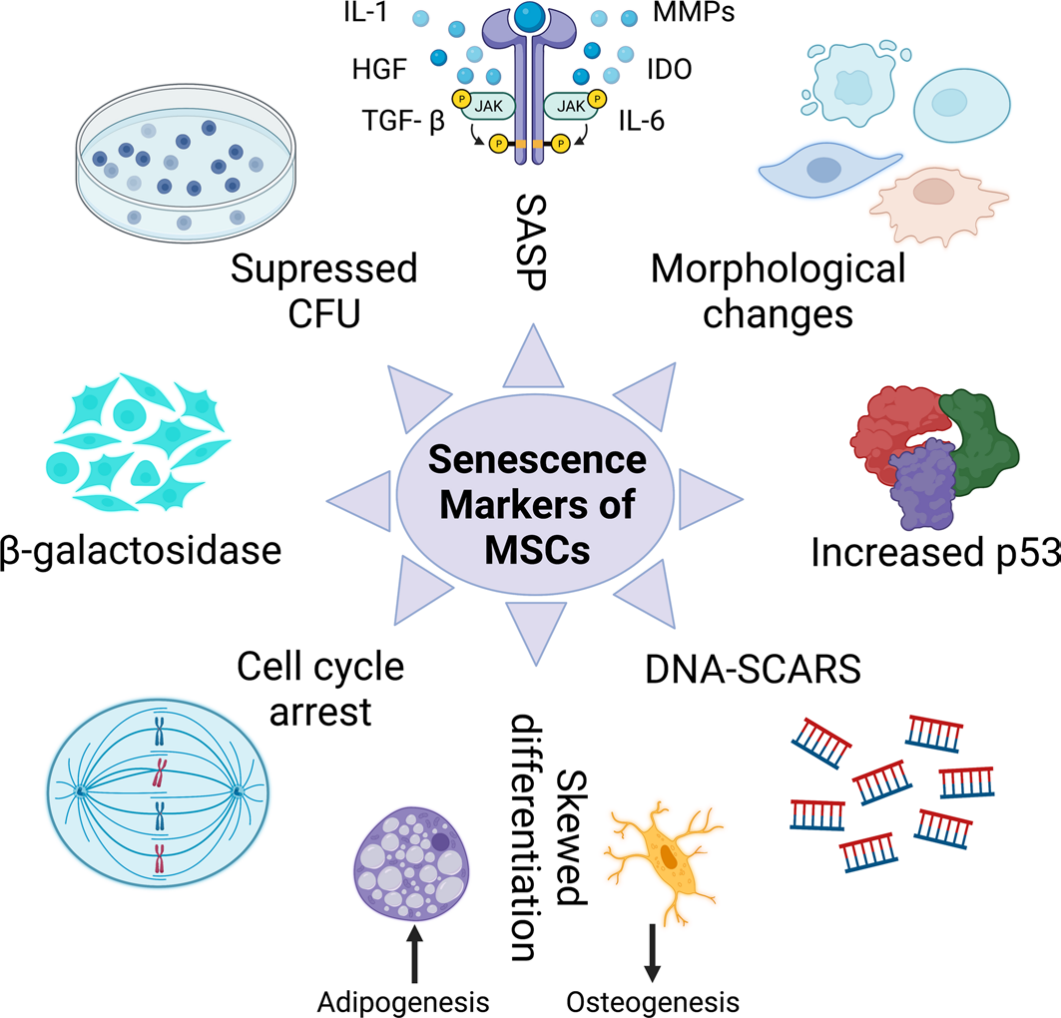
Overview of replicative senescence markers in MSCs. The major markers of MSC aging in laboratory are DNA damage, P53 upregulation,  SA-β-gal expression, morphological changes, cell-cycle arrest, skewed differentiation, induced SASP, and compromised colony-forming ability. Activate (  )

**Fig. 5:**
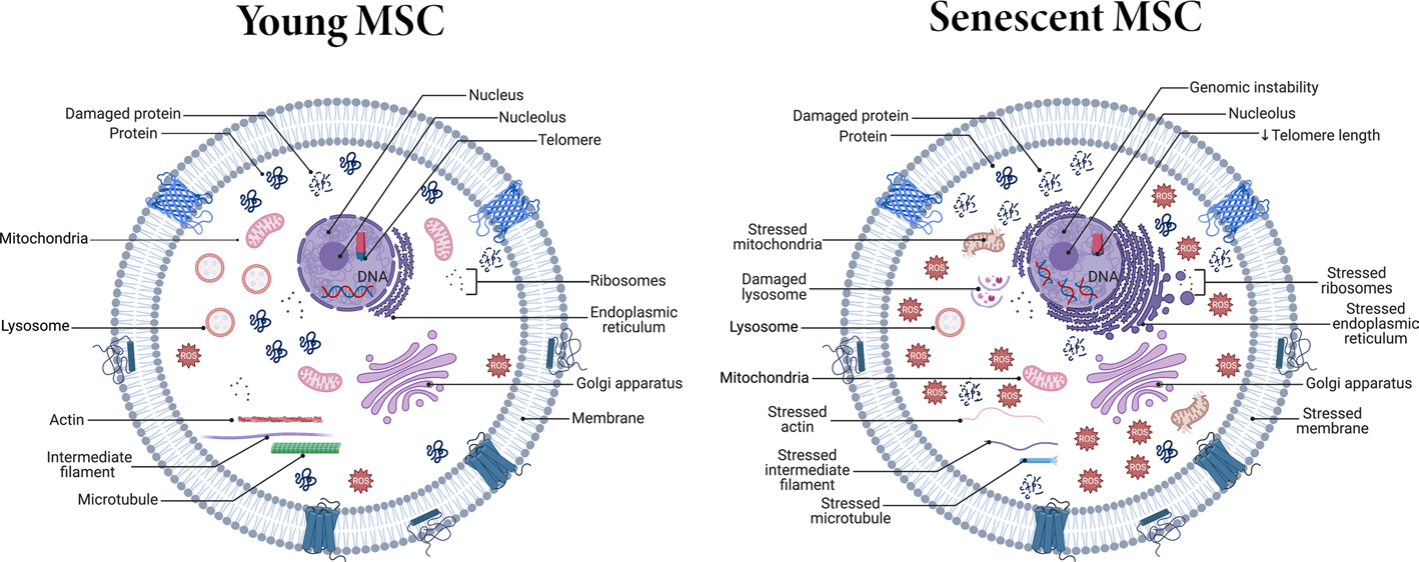
Concept of young MSCs versus senescent MSCs, showing the major cellular and organellar differences between normal and senescent MSCs. Senescent MSCs contain more damaged DNA and proteins, stressed organelles, short telomeres, and induced ROS

After explaining the mechanisms that orchestrate the senescence of MSCs, it is important to enumerate the laboratory signs that characterize aged MSCs. Herein we discuss the major markers of MSCs’ replicative senescence (Fig. 4) and try to show a concept for young and senescent MSCs (Fig. 5).

#### Morphological changes

Normal morphology of MSCs in cell culture is characterized by spindle shape with a small cell body and a few long thin processes as well as a large nucleus with a differentiated nucleolus. However, the morphology of aged MSCs usually changes to become more enlarged, lose its spindle-shaped characteristics, and flatten. Of note, a long period of in vitro expansion-dependent senescence of adipose tissue MSCs revealed morphological changes, including increased size and shape complexity giving a fried-egg-like appearance [225]. Researchers have tried to identify a procedure that maintains the normal morphology of MSCs. For example, polycarbonate substrate was recommended in cell culture plates because it promoted MSC longevity and spindle-shaped cells compared with MSCs cultivated in polystyrene substrate [226].

#### Upregulation of the P53 pathway

As we mentioned above, the primary marker for MSC senescence is increased expression of p53 and its related proteins, such as p16^Ink4a^ and p21. In other word, MSCs cannot be considered senescent if the expression of p53 is at normal levels. The components of the p53 pathway are becoming commonly used as aging markers for cellular senescence along with beta-galactosidase upregulation [227, 228].

#### DNA-SCARS

When MSCs enter senescence mode, one of the major mechanisms activated is DNA damage, which in turn produces DNA- segments with chromatin alterations reinforcing senescence (SCARS). The DNA damage during MSC senescence is discussed above in detail. It has been reported that oxidative-stress-dependent DNA damage is one of the markers of MSC senescence [229].

#### Skewed differentiation

One of the most important characteristics of MSCs’ stemness is normal differentiation, the tendency of MSCs’ differentiation capacity toward osteocytes but not adipocytes. Biased differentiation occurs when this tendency changes toward adipocytes and decreases toward osteocytes. Because this case is a prominent sign of MSC senescence, researchers have been trying to identify procedures that contribute to correcting the case of senescent MSCs with biased differentiation to improve the treatment of patients with aging-induced bone loss such as osteoporosis. For example, scientists found that microRNA-10b had a positive effect in regulating the balance of osteogenesis and adipogenesis differentiation of MSCs from adipose tissues through TGF-β/SMAD2. This discovery may introduce a potential tool for improving impaired osteogenesis [230].

#### Cell cycle arrest

The accumulation of MSCs at any phase of cell cycle is an indication of molecular defect of aging-related signaling pathways, such as p53 and PI3K/Akt/mTOR pathways, which regulate MSCs during mitosis. Thus, cell cycle arrest is considered a marker of MSC senescence. It is reported that senescent MSCs presented G0/G1 cell cycle arrest in association with other prominent senescence markers [231]. Meanwhile, blebbistatin-induced senescence of MSCs from Wharton’s jelly presented G0/G1 cell cycle arrest associated with increased expression of cell cycle inhibitors CDKN1A and CDKN2A [232]. Also, the well-known senescence inducer H_2_O_2_ stimulated G0/G1 cell cycle arrest [233].

#### Beta-galactosidase (β-gal)

β-Gal is an enzyme that hydrolyzes β-galactosides into monosaccharides. It is a well-known tool to identify aged cells in laboratory cell culture after being stained by immunostain specific to β-gal protein to give light-green color. Scientists can consider β-gal upregulation as a senescence marker in MSCs [234].

#### Suppressed colony-forming ability

Colony formation is an indicator of MSCs’ stemness. When MSCs lose the capacity to form colonies, this is a sign of decreased proliferation and induced senescence. Colony-forming units (CFUs) were used to determine the optimum growth rate of MSCs during in vitro expansion [235]. As well, CFU could be considered as an indicator in tracking and follow-up of replicative senescence of MSCs [236].

#### SASP

In senescence, MSCs display specific secretions that regulate and maintain the aging phenotype. These secretions include IL-6, IDO, TGF- β, HGF, and a variety of secretory cytokines and chemokines. It is proven that SASP enables senescent cells to participate in remodeling their environment through modulation of multiple physiological functions including wound healing, cancer suppression, and embryonic development. SASP is also associated with increased expression of proteases and metalloproteinase (MMPs) that may affect the extracellular matrix [237]. In addition, secretory cytokines, growth factors, and proteinases of senescent MSCs are reported to be not only aging markers but also aging triggers in senescence of MSCs derived from human endometrium [238]. Indeed, SASP from MSCs of bone marrow and adipose tissues were analyzed and reported with signaling ability to maintain and induce senescence in their niche [239]. Interestingly, the underlying mechanism that regulates SASP in MSCs was attributed to *GATA4*, which mediates MCP-1 expression in progerin or/and prelamin-dependent pathways [240]. Also, Hisamatsu et al. reported that the young MSC secretome contains growth differentiation factor 6, which may play an important role in regulating the effects of old-MSC' SASP factors in geriatric diseases [241]. More importantly, MSCs from bone marrow can be cannibalized with other cancerous cells to promote tumor dormancy and SASP factors that contribute to the evolution of tumor recurrence [242]. Altered paracrine secretion was associated with IHH depletion-induced senescence in BMSCs. This secretion includes upregulation of IL-6, IDO, and COX2, and downregulation of TGF-β and HGF [123]. This finding indicates that BMSC senescence is characterized by specific SASP that may stimulate senescence phenotype through cell-to-cell contact using the above-mentioned proteins of SASP .

### Prospected strategies to avoid or combat cellular senescence

**Fig. 6:**
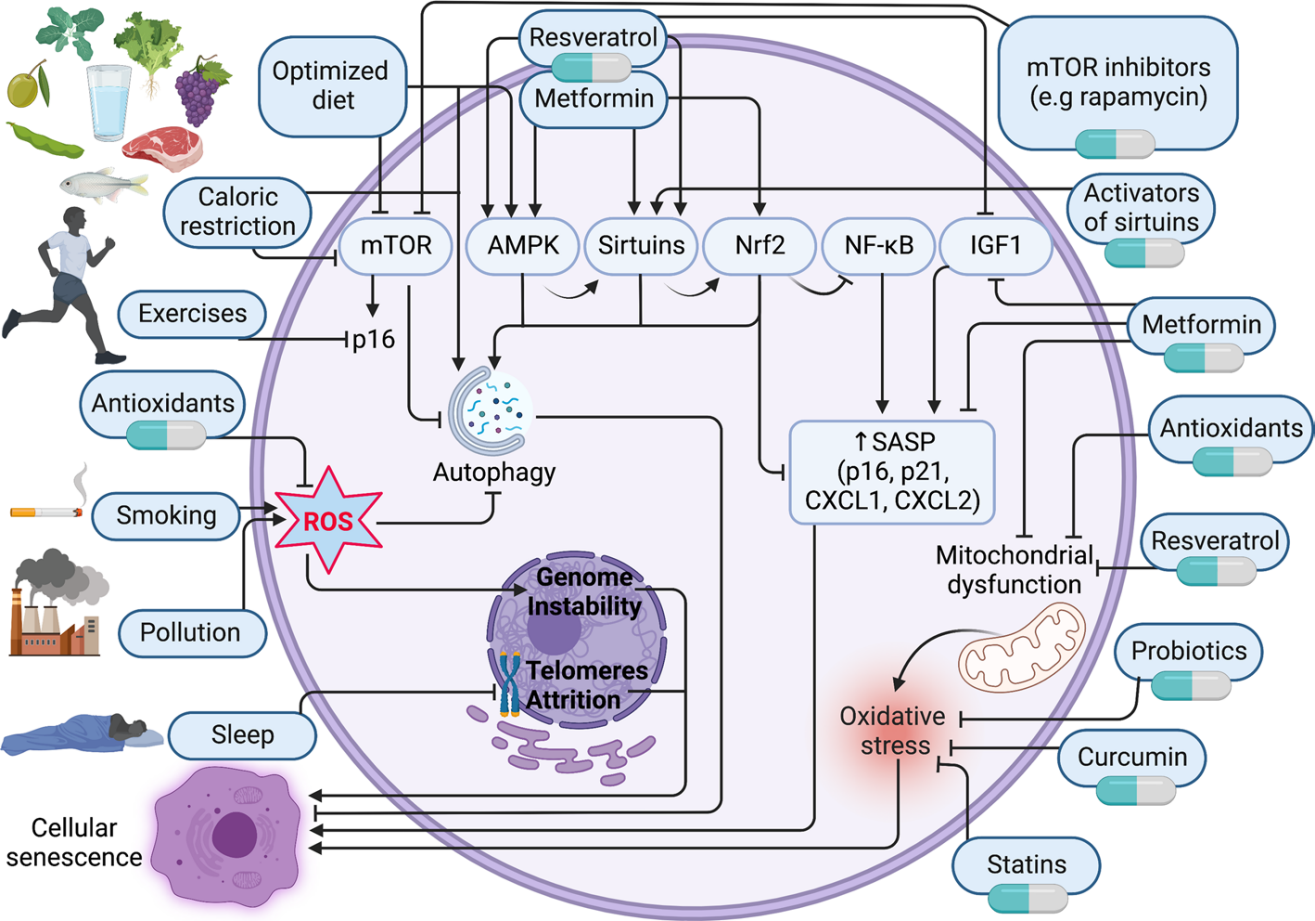
Lifestyle modifications and pharmacological interventions are strategies to combat aging and cellular senescence. Optimizing lifestyle with appropriate diet, exercise, sleep, and no smoking and pollution is critical to avoiding aging and cellular senescence. Pharmacological interventions by metformin, resveratrol, curcumin, statins, antioxidants, mTOR inhibitors, sirtuins activators, caloric restriction mimetics, and probiotics are also suggested as anti-aging remedies.  Activate,  inhibit

**Fig. 7:**
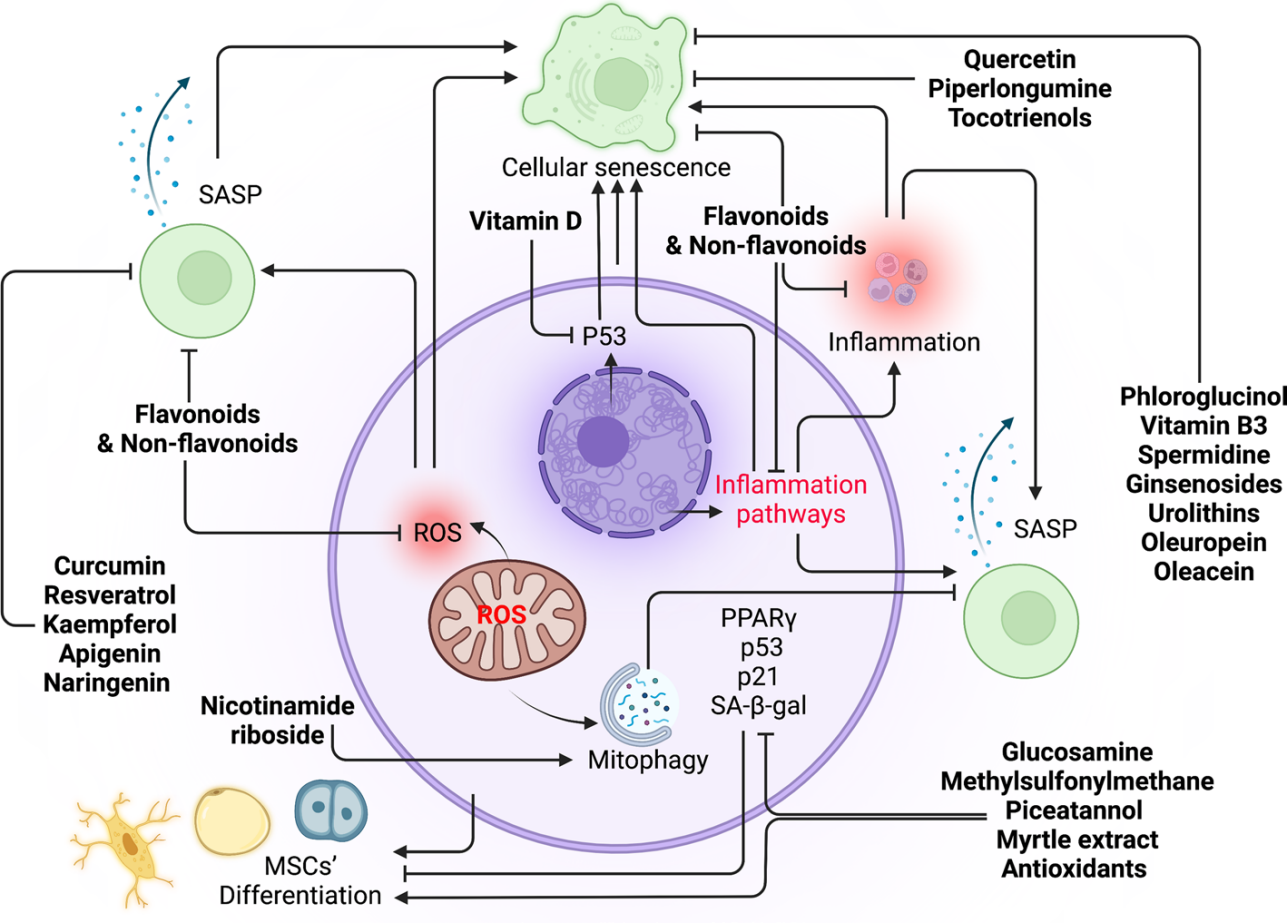
Nutraceutical intervention as a strategy to combat cellular senescence. Flavonoids, nonflavonoids, and other nutraceuticals compromise cellular senescence through regulating ROS, inflammation, SASP, p53, p21, and PPARγ.  Activate,  inhibit, nutraceuticals (bold)

**Fig. 8:**
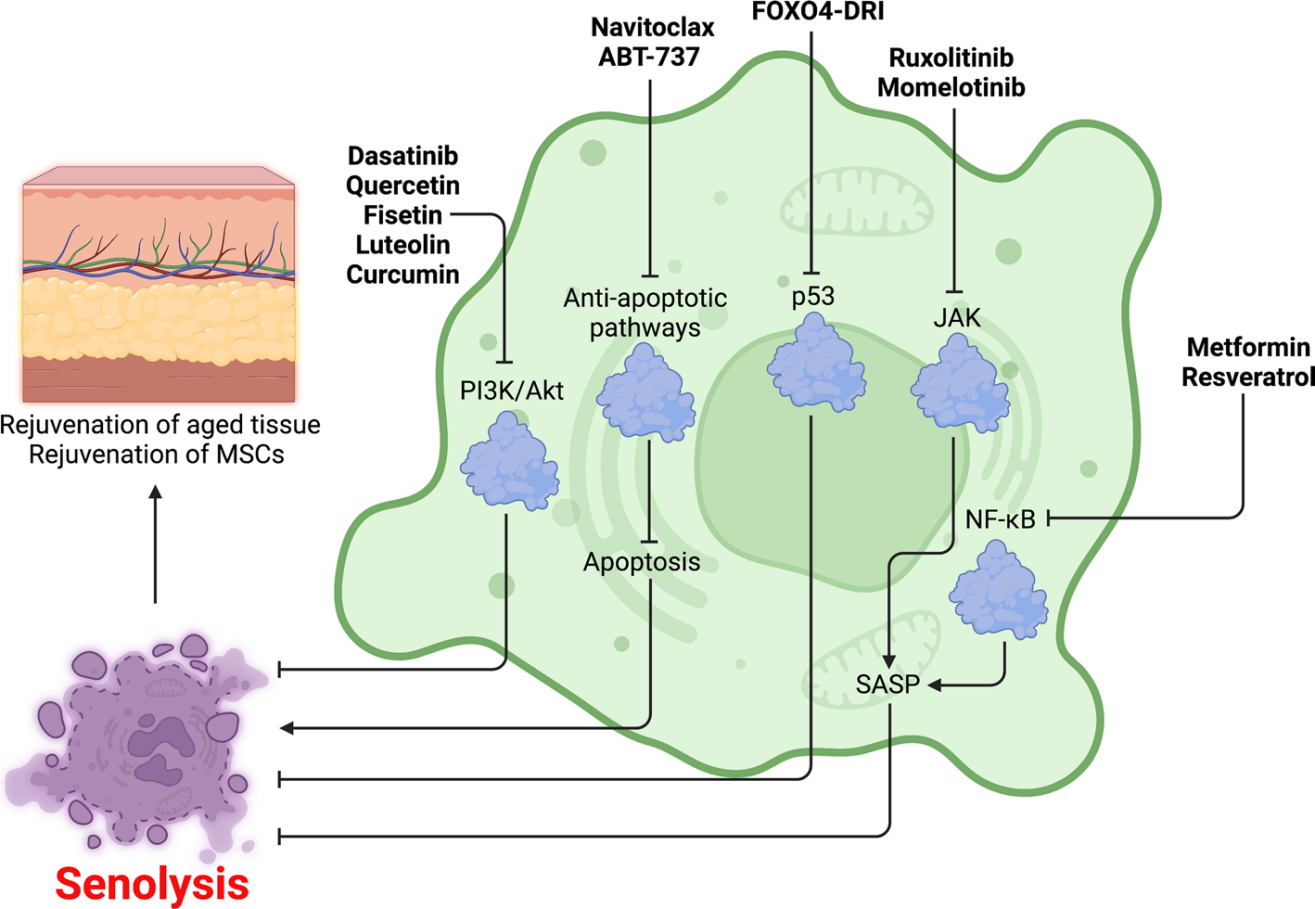
Senolysis as a strategy to combat cellular senescence. Indicated senolytics induce cellular rejuvenation through targeting anti-apoptotic pathways, and PI3K/Akt, p53, NF-κB, and JAK signaling pathways.  Activate,  inhibit, senolytics (bold)

As we explained above, cellular senescence is an intricate biological phenomenon that can be regulated by the overlap of many factors. Therefore, scientific efforts focus on finding ways that can interfere with cellular senescence inducers to produce anti-aging criteria that can be followed. Though there are many interplaying regulators for cellular senescence, here we discuss the three major outlines strategies that in turn include many directions for each: modification of lifestyle, pharmacological (Fig. 6) and nutraceutical (Fig. 7) interventions, and senolytic drugs (Fig. 8).

#### Lifestyle modifications

Physical activity, nutritional system habits, sleep, and environmental factors, e.g., smoking and air pollution, are the main components of lifestyle that can be considered in combating cellular senescence (Fig. 6).

Physical exercise is a collection of planned and repetitive movements for the whole body or some body regions that oppose the sedentary living lifestyle. Physical exercise is already known to have anti-aging effects, via stimulation of anti-aging pathways, AMPK, and sirtuins [243, 244]. Exercise can induce mesenchymal and neural stem cell migration and differentiation. The antisenescence effects of exercise are attributed to their role in promoting the length of telomeres and decreasing p16^Ink4a^ and p53 expression [244]. A newly published report suggests that a 12-week  structured exercise program can compromise cellular senescence parameters p16, p21, cGAS, and TNF-α [245]. Preclinical data revealed that the treatment of degenerative neural diseases by stem cell transplantation can be enhanced by physical exercise [246]. Exercise also has a positive role in inducing muscle regeneration through inducing fibro-adipogenic progenitor senescence [247]. As MSCs are mechanosensitive, exercise can stimulate the molecular machinery of longevity and prevent some aging-related conditions, including osteoporosis and obesity [248].

Nutritional habits are also related to cellular senescence; for example, high caloric intake can be sensed by nutrient-sensing pathway, IIS. Therefore, dietary interventions by caloric restriction, different types of fasting, diets with no or low/high quantity of some nutrients, fatty acids, and phytochemicals, or time-restricted eating may have anti-cellular senescence effects. A recent report stated that caloric restriction promotes antisenescence action by upregulation of lncRNA-KCNQ1OT1-MIR-760 [249]. The underlying mechanism of this is that caloric restriction increases telomerase activity [250] and induces protein kinase (CK2) expression, which in turn activates AMPK, sirtuins, and autophagy [251]. Collectively, caloric restriction prevents stem cell aging through maintaining their cellular and acellular niche, enhancing their proliferation and self-renewal activity [252]. In the same context, intermittent fasting, fasting-mimicking diet, time-restricted feeding, and alternate-day fasting can contribute to improved health parameters and induce longevity through IIS downregulation. It is also proposed that practicing a diet with a restricted quantity of specific nutrients may promote longevity; for example, restriction of monosaccharides or amino acid methionine may modulate senescence via Mtorc1 modulation. There is no doubt that fresh vegetables, fruits, some grains and pulses, protein-rich food, fish, and olive oil, which contain vitamins (A, E, C), as well as fibers, minerals, essential polyunsaturated fatty acids, and phytochemicals (phytosterols, polyphenols, terpenoids, and carotenoids), can combat aging. Hormesis concept is considered as one of the anti-aging effects of these nutrients that induces an interplay of responses contributing to the process of longevity. Among the suggested mechanisms for this hormetic effect is inhibition of NF-κB, modulation of mTOR, and activation of sirtuins and Nrf2 [253]. More interestingly, polyphenols can prevent cellular senescence by targeting microRNA, inhibiting mitochondrial dysfunction, and downregulating ROS [254].

One important component of lifestyle is sleep behavior which is closely related to MSCs stemness because of melatonin, the “sleep hormone.” It is reported that melatonin has an antireplicative senescence effect and can induce MSC proliferation and immunomodulatory potency [255, 256]. In addition, delayed sleep is associated with telomere shortening, one of the hallmarks of aging [257]. Indeed, single-cell RNA sequencing for immune cells revealed that poor sleep compromised immune cell differentiation and induced cellular senescence [258]. Thus, having a sufficient quantity and quality of sleep during the nighttime could avoid and/or reverse cellular senescence owing to the availability of melatonin.

Smoking is also reported to have a role in inducing cellular senescence markers in lung cells, indicating the importance of stopping this habit to prevent aging [259]. Another important environmental factor that can activate cellular senescence is air pollution. It is reported that some pollutants in Brazil, China, and South Africa may predispose cellular senescence by inducing telomere shortening or reducing catalase activity [260–262]. Furthermore, endothelial cells, skin keratinocytes, and mouse lung fibroblast can enter cellular senescence mode because of pollutants, fine dust, particulate matter 2.5, and polycyclic aromatic hydrocarbons via inducing senescence regulators, ROS, or the ATM serine/threonine kinase/H2A histone family member X pathway [263–265]. Therefore, staying away from air pollution and keeping our environment clean is a crucial component of anti-aging strategies.

#### Pharmacological and nutraceutical interventions

On the basis of our current understanding of aging and cellular senescence, the most obvious therapeutic strategies to avoid or/and reverse this phenomenon are (a) inhibition of ROS and/or mTOR, (b) caloric restriction mimetics, (c) activation of AMPK, sirtuins, and/or Nrf2, (d) targeting of SASP pathways, (e) secured gut microbiota homeostasis [266], and (f) senolysis, i.e., removal of senescent cells [267]. Additionally, targeting of *JAK/STAT, cGAS-STING,* and NF-κB signaling may compromise cellular senescence by modulating SASP [268].

Here we discussed briefly the most important pharmacological interventions that can induce cellular longevity (figure 6). For example, SOD and catalase inhibited oxidative-stress-induced MSC senescence and induced osteogenesis [269]. Antioxidant, NAC also suppresses BMSC senescence and skewed differentiation through maintaining genomic stability, telomere length, and telomerase activity [270]. In addition, ferulic acid reversed stem cell senescence in an antioxidant-dependent manner in mice exposed to whole-body irradiation [271]. Indeed, a novel promising antioxidant intervention targeting epigenetic regulator EZH2 can promote BMSC longevity [272]. Please refer to a review containing a comprehensive explanation for antioxidants used for MSCs’ stemness and longevity including all kinds of antioxidants (chemical compounds, biometabolites, and proteins or precursors)  with their mode of action [273].

In experimental animals, inhibition of mTOR by rapamycin is promising in the treatment of ischemic diseases because of its ability to reverse transplanted human MSC senescence [274]. It is explained that rapamycin can exert this anti-aging effect through suppressing p16^Ink4a^ accumulation [275]. Metformin also compromised dental pulp stem cell senescence by downregulation of microRNA-34a-3p through activation of AMPK and inhibition of mTOR phosphorylation [276]. It is clear that pharmacological intervention by targeting mTOR using rapamycin and its derivatives or other mTOR inhibitors can contribute to stem cell cellular longevity.

Nowadays, one of the most common activators of AMPK and sirtuins is resveratrol, a polyphenol found in grapes. Resveratrol can induce longevity through modulation of oxidative stress, inflammation, nutrient-sensing pathways, and maintenance of telomeres [277]. Thus, resveratrol is suggested to be used as an anti-aging agent and in the management of some aging-related diseases [278].

Because metformin is clinically approved for diabetes mellitus type 2, we think that, to date, it is the golden-standard anti-aging and anti-cellular senescence drug, but it still needs further clinical optimization to be prescribed as an anti-aging drug. Metformin can attenuate aging through activating AMPK, sirtuins, autophagy, mitochondrial biogenesis, and Nrf2. In the meantime, metformin can inhibit IIS, modulate epigenetic alterations, and prevent DNA damage and telomere attrition [170]. In the same context, using caloric restriction mimetic agents is promising as an anti-aging remedy through improving nutrient-sensing signaling, AMPK, sirtuins, and IIS in a hormetic-dependent manner. For example, activating autophagy through 3,4-dimethoxychalcone stimulates transcription factors E3 and EB [279]. On the other hand, vegetables and seaweeds as a resource for extracting bioactive molecules to be used as anti-senescence agents have also been reviewed [280, 281]. Curcumin is also reported to have an anti-aging role in the treatment of aging-related diseases, cancer, and arthritis [282]. In addition, pharmaceutical activator of sirtuins, resveratrol, curcumin, statins, melatonin, cilostazol, hydrogen sulfide paeonol, icariin, persimmon, and NAD^+^ activators, are promising to prevent cellular senescence [283, 284]. For instance, resveratrol inhibited MSC senescence through regulating reticuloendotheliosis viral oncogene homolog A (RELA)/sirtuin-1 pathway [285].

The involvement of gut microbiota dysbiosis in aging biology is already reported, indicating the importance of keeping stable gut microbiota’s niche as an anti-aging strategy. Thus, the use of probiotics is useful to inhibit the aging process through modulating the immune response, antioxidant defense, and sirtuins [286]. The effects of anti-aging interventions on the population and environment of gut microbiota were discussed in this paper [287]. Probiotics were also considered as anti-aging effectors in skin issues and dermatology [288].

Using the above-mentioned food-derived compounds as anti-cellular senescence agents led to the study of a wide variety of nutraceuticals (Fig. 7), including dietary supplements and functional food to be used in fighting against cellular senescence and aging-related diseases [289]. Nutraceutical compounds are biomolecules that are found naturally in food or other natural resources, and some of them may have antisenescence effects [290, 291]. For instance, quercetin, a flavonol found in some fruits; piperlongumine, naturally found in *Piper longum*; and tocotrienols, part of the vitamin E family, all have senolytic effects on senescent cells [292–295]. In addition, there is a vast group of nutraceuticals called polyphenols that can exert antioxidant and anti-inflammatory actions, thereby promoting antisenescence pathways. Polyphenols encompass thousands of compounds found in food, especially fruits and vegetables. They are classified into two broad categories, flavonoids and nonflavonoids, and they have been shown to have anti-SASP effects through downregulation of oxidative stress and inflammation pathways [254, 291]. Although the roles of polyphenols in cellular senescence are not fully investigated, it is reported that some of them, including curcumin, resveratrol, kaempferol, apigenin, and naringenin, are potentially have antisenescence roles. Phenol-Explorer (http://phenol-explorer.eu/compounds) is a database that contains details about different sources and properties of polyphenols. Meanwhile, other bioactive compounds found in food, phloroglucinol, vitamin B3, spermidine, ginsenosides, urolithins, oleuropein, and oleacein are also suggested to have a putative antisenescence effects [291]. In the same context, nicotinamide riboside stimulated antisenescence phenotype and downregulated SASP by promoting mitophagy in a PTEN-induced kinase 1-dependent manner [296]. Nicotinamide mononucleotide induced anti-aging miRNA expression profile in the aorta of aged mice [297]. Creatine, an amino acid found in meat and seafood, can inhibit senescence in rats with doxorubicin-induced liver injury [298]. Vitamin D can prevent the progression of nonalcoholic fatty liver disease through inhibiting cellular senescence of hepatocytes, inducing antioxidant pathways, and downregulating p53 [299]. A clinical trial showed that vitamin D induced a significantly increased count of circulating osteoprogenitor cells [300]. Accordingly, translational research of nutraceuticals may introduce specific anti-aging agents for different aging-related diseases [301]. In closing, using nutraceuticals as antisenescence agents is a promising path toward finding a novel strategy for fighting aging-related diseases.

As they have effects on cellular senescence, nutraceuticals may also have anti-aging effects in MSCs. It has been demonstrated that MSCs’ stemness could be promoted by some food-derived nutrients. For example, glucosamine, an amino monosaccharide, induced human MSC chondrogenesis through downregulation of metalloproteinase 13 [302]. Similarly, a nutraceutical compound, methylsulfonylmethane promoted MSC differentiation, chondrogenesis, and preosteoblast formation [303]. In addition, a mixture of 36 nutrients promoted proliferation and osteogenic differentiation and inhibited adipogenesis of BMSCs from rats with aplastic anemia [304]. Indeed, piceatannol inhibited adipogenic activity in human MSC-derived adipocytes through PPARγ downregulation [305]. At the same context, using honey silk fibroin scaffold decreased the expression of MSC senescence markers p53, p21, and SA-β-gal [306]. Moreover, myrtle extract from *Myrtus communis* L. was reported to have antisenescence effects on stem cells of the skin and adipose tissue [307]. Furthermore, supplemented antioxidants are reported as cytoprotective agents for MSCs that may induce their therapeutic potency [273]. Taken together, the issue of nutraceutical and MSC senescence still needs more investigation to be translated into research and then the clinical field.

#### Senolysis and senolytic drugs

Senolysis is the process of removing senescent cells from normally proliferative cells’ niche using specific agents that selectively clear them. These agents are called senolytic drugs, which can clear apoptosis-resistant senescent cells through inducing their apoptosis pathways. Although use of senolytic drugs in the clearing of senescent cells is still in the preclinical and clinical stages of research, translation of these findings to the clinical field is a promising and hot topic owing to their predictive role in the treatment of a wide variety of aging-related diseases [212, 308, 309]. Many groups of senolytic drugs are used to combat cellular senescence at the level of research (Fig. 8). These groups include epigenetics-dependent rejuvenation agents, senoblockers; SASP inhibitors, senomorphics; SASP suppressors, senomodulators; drugs stopping cellular senescence initiation, senostatics; and molecules delaying senescent cell accumulation rate, senosuppressors. The mode of action for senolytic drugs is summarized by targeting senescence-related pathways, including anti-apoptotic pathways, p53, p16, NF-κB, PI3K, and others [310, 311]. For example, dasatinib, quercetin, fisetin, luteolin, and curcumin target the PI3K/Akt pathway; navitoclax and ABT-737 target anti-apoptotic pathways; FOXO4-DRI target the p53 pathway; metformin and resveratrol target the NF-κB pathway; and ruxolitinib and momelotinib target the JAK pathway [310, 312]. Many aging-related diseases have benefited from the use of senolytic drugs at the preclinical stage in experimental animals [313, 314]. Three reviews present a detailed discussion about the diverse anti-cellular senescence aspects of senolytic drugs [310, 313, 315].

In relation to stem cells, oral administration of senolytic drug ABT263 in mice contributed to the rejuvenation of aged tissue stem cells, including hematopoietic stem cells and muscle stem cells, through inducing apoptosis in senescent stem cells [316]. Quercetin is effective at removing senescent BMCSs of mouse [212]. Metformin has also a senomorphic effect on MSCs through anti-ROS action, thereby inhibiting replicative senescence [317]. It is reported that dasatinib can target senescent MSCs from adipose tissue of patients with preeclampsia through decreasing SASP and p16 [318]. Consistently, senolytic mixture of quercetin and dasatinib, D + Q, activates osteogenic potency of BMSCs in vitro and in vivo [319]. On the other hand, senolytic drugs danazol, nicotinamide riboside, quercetin, and ABT-263 were tested for their effects on human MSCs, and none of them except ABT-263 had a senolytic effect by decreasing SA-β-gal, and had no effect on proliferation, length of telomeres, or epigenetic alterations [71]. Collectively, senolytics in anti-cellular senescence, in general are promising and moderately investigated, but in MSCs senescence still requires intensive study.

## Conclusion

In vivo senescence of MSCs is part of the human aging phenomenon, which is one of the underlying causes of aging-related diseases. Additionally, owing to their immunomodulatory potency, MSCs are now the backbone of cellular therapy in the management of many diseases such as autoimmune diseases, degenerative diseases, and other aging-related diseases at the level of clinical trials. Sadly, MSC senescence in vitro or in vivo is a major challenge in the field of cellular therapy in which senescent MSCs develop SASP, which changes the characteristics of their therapeutic potency. These changes can cause life-threatening risks, including tumorigenesis and adverse immune stimulation, through the secretome of SASP components. Nowadays, scientists have successfully identified promoters of MSC aging such as genetic martial deterioration, noncoding RNA, exosomes, protein imbalance, mitochondrial dysfunction, and  mTOR, ROS, and IIS signaling pathways. Indeed, they detected anti-aging signaling pathways such as AMPK, sirtuins, Nrf2, and Hedgehog. Moreover, intervention strategies for reversing and/or avoiding cellular senescence will be elucidated over time. These directions include lifestyle modification, antioxidants, mTOR inhibitors, NF-κB inhibitors, activators of AMPK, sirtuins, and Nrf2, improving nutrient-sensing signaling, modulating SASP, nutraceutical interventions, senolytic drugs, and probiotics. This knowledge about aging can be a very useful tool for researchers to identify the molecules that orchestrate aging homeostasis. Exploring these molecules may introduce solutions for humanity to fight aging-related diseases and improve cellular therapy.

## Data Availability

Not applicable.

## Abbreviations

AMPK: Adenosine monophosphate-activated protein kinase
AP2: Adipocyte protein 2
bFGD: Basic fibroblast growth factor
c–c motif: Chemokine
CCR2: Receptor 2
E2: Estradiol 2
ERR α: Estrogen-related receptor α
ERK: Extracellular signal-regulated kinase
FOXO3a: Forkhead box O3a
Hsp72: Heat shock protein 72
HIV: Human immunodeficiency virus
MSCs: Human mesenchymal stem cells
hTERT: Human telomerase reverse transcriptase
IIS: Insulin/IGF-1-like signaling
IGF1: Insulin-like growth factor 1
LPS: Lipopolysaccharide
LPL: Lipoprotein lipase
mTOR: Mammalian target of rapamycin
MMPs: Metalloproteinase
MIF: Migration inhibitory factor
MRC: Mitochondrial respiratory chain
MAPK: Mitogen-activated protein kinases
MCP1: Monocyte chemoattractant protein 1
NDNF: Neuron-derived neurotropic factor
Nampt: Nicotinamide phosphoribosyl transferase
NF-κB: Nuclear factor kappa-light-chain-enhancer of activated B cells
OS: Oxidative stress
PPARγ: Peroxisome proliferator-activated receptor gamma
PI3k: Phosphatidylinositol 3-kinase
PEDF: Pigment epithelium-derived factor
PDGF: Platelet-derived growth factor
PGC-1α/β: PPARγ co-activator 1α/β
ROS: Reactive oxygen species
SERCA: Sarcoplasmic/endoplasmic reticulum calcium-ATPase
SASP: Senescence-associated secretory phenotype
STAT3: Signal transducer and activator of transcription 3
SOD2: Superoxide dismutase 2
MnSOD: Superoxide mutase
SLE: Systemic lupus erythematosus
Nrf2: Nuclear factor erythroid 2-related factor 2
